# Glucocerebrosidase Deficiency in *Drosophila* Results in α-Synuclein-Independent Protein Aggregation and Neurodegeneration

**DOI:** 10.1371/journal.pgen.1005944

**Published:** 2016-03-28

**Authors:** Marie Y. Davis, Kien Trinh, Ruth E. Thomas, Selina Yu, Alexandre A. Germanos, Brittany N. Whitley, Sergio Pablo Sardi, Thomas J. Montine, Leo J. Pallanck

**Affiliations:** 1 Department of Neurology, University of Washington, Seattle, Washington, United States of America; 2 Department of Genome Sciences, University of Washington, Seattle, Washington, United States of America; 3 Department of Biochemistry, University of Washington, Seattle, Washington, United States of America; 4 Neuroscience, Rare Diseases Research Unit, Sanofi Genzyme, Framingham, Massachusetts, United States of America; 5 Department of Pathology, University of Washington, Seattle, Washington, United States of America; Stanford University School of Medicine, UNITED STATES

## Abstract

Mutations in the *glucosidase*, *beta*, *acid* (*GBA1*) gene cause Gaucher’s disease, and are the most common genetic risk factor for Parkinson’s disease (PD) and dementia with Lewy bodies (DLB) excluding variants of low penetrance. Because α-synuclein-containing neuronal aggregates are a defining feature of PD and DLB, it is widely believed that mutations in *GBA1* act by enhancing α-synuclein toxicity. To explore this hypothesis, we deleted the *Drosophila GBA1* homolog, *dGBA1b*, and compared the phenotypes of *dGBA1b* mutants in the presence and absence of α-synuclein expression. Homozygous *dGBA1b* mutants exhibit shortened lifespan, locomotor and memory deficits, neurodegeneration, and dramatically increased accumulation of ubiquitinated protein aggregates that are normally degraded through an autophagic mechanism. Ectopic expression of human α-synuclein in *dGBA1b* mutants resulted in a mild enhancement of dopaminergic neuron loss and increased α-synuclein aggregation relative to controls. However, α-synuclein expression did not substantially enhance other *dGBA1b* mutant phenotypes. Our findings indicate that *dGBA1b* plays an important role in the metabolism of protein aggregates, but that the deleterious consequences of mutations in *dGBA1b* are largely independent of α-synuclein. Future work with *dGBA1b* mutants should reveal the mechanism by which mutations in *dGBA1b* lead to accumulation of protein aggregates, and the potential influence of this protein aggregation on neuronal integrity.

## Author Summary

Mutations in the *glucosidase*, *beta*, *acid* (*GBA1*) gene cause Gaucher’s disease (GD), a lysosomal storage disease that includes neurodegenerative phenotypes. Recently, mutations in *GBA1* were identified as the strongest genetic risk factor for Parkinson’s disease (PD) and dementia with Lewy bodies (DLB), which are neurodegenerative conditions characterized by intraneuronal protein aggregates containing α-synuclein. To explore how *GBA1* mutations lead to neurodegeneration in GD, PD and DLB, we developed a novel invertebrate model of *GBA1* insufficiency by deleting the *Drosophila GBA1* homolog, *dGBA1b*. We found that *dGBA1b* mutants have multiple phenotypes consistent with neuronal dysfunction as seen in PD, DLB and GD, and dramatically increased protein aggregation that is normally cleared through an autophagic mechanism. *dGBA1b* mutants expressing human α-synuclein in dopaminergic neurons led to dopaminergic neuron loss and α-synuclein aggregation. However, α-synuclein expression had minimal effect on *dGBA1b* mutant phenotypes, suggesting that the deleterious consequences of glucocerebrosidase deficiency are independent of α-synuclein. These findings significantly contribute to our understanding of the role of *GBA1* mutations in the pathogenesis of PD, DLB, and GD, and further studies using this model should elucidate mechanisms underlying these diseases.

## Introduction

Gaucher’s disease (GD), the most common lysosomal storage disorder, is caused by recessive mutations in the *glucosidase*, *beta*, *acid 1* (*GBA1*) gene, which encodes the lysosomal enzyme glucocerebrosidase [1, 2]. GD has traditionally been categorized into neuronopathic and non-neuronopathic subtypes. However, recent work has challenged this classification by revealing that both subtypes are associated with a significantly elevated risk of Parkinson’s disease (PD) [3–6], the most common neurodegenerative movement disorder. PD is characterized pathologically by the degeneration of dopaminergic neurons in the midbrain and the accumulation of ubiquitinated intraneuronal protein aggregates called Lewy bodies. More recently, heterozygous mutations in the *GBA1* gene were found to be among the most common genetic associations with sporadic PD and dementia with Lewy bodies (DLB) [7–9]. The mechanism by which mutations in *GBA1* cause these neurodegenerative disorders is currently unclear.

A number of different models have been proposed to explain the influence of *GBA1* mutations on PD, DLB and neuronopathic forms of GD, with α-synuclein protein playing a prominent role in many of these models [3, 4, 10, 11]. α-synuclein is a major component of the Lewy body aggregates that define sporadic PD and DLB, and mutations that lead to increased expression of α-synuclein cause heritable forms of PD with a disease severity commensurate with α-synuclein abundance [12, 13]. The finding that glucocerebrosidase normally localizes to the lysosome has led to the model that mutations in *GBA1* impair the lysosomal degradation of misfolded forms of α-synuclein, resulting in toxic accumulation of α-synuclein aggregates. While previous work offers support for the model that *GBA1* mutations trigger the accumulation of α-synuclein aggregates [14, 15], the mechanism by which they do so remains controversial. Moreover, the extent to which α-synuclein contributes to the pathogenesis of *GBA1*-associated neurodegenerative diseases also remains unclear.

To explore the mechanism by which mutations in *GBA1* lead to neurodegenerative diseases, we created a *Drosophila* model of glucocerebrosidase deficiency by generating a deletion of the *Drosophila GBA1* homolog *dGBA1b*. *Drosophila dGBA1b* mutants exhibit shortened lifespan, locomotor, memory and other behavioral deficits, neurodegeneration, and accumulation of insoluble protein aggregates that are normally degraded through an autophagic process. While glucocerebrosidase deficiency mildly enhanced the toxicity of α-synuclein in dopaminergic neurons, and resulted in increased α-synuclein aggregation, α-synuclein expression did not enhance any of the phenotypes of *dGBA1b* mutants. Together, our findings indicate that mutations in *GBA1* lead to an increased abundance of proteins normally degraded by autophagy, but that the phenotypes accompanying glucocerebrosidase deficiency are largely independent of α-synuclein. Future studies of *Drosophila dGBA1b* mutants should clarify the nature of this autophagic defect, and will provide further insight into the pathogenesis of GD, PD, and DLB.

## Results

### Glucocerebrosidase deficiency in *Drosophila* results in shortened lifespan and behavioral deficits

**Fig 1:**
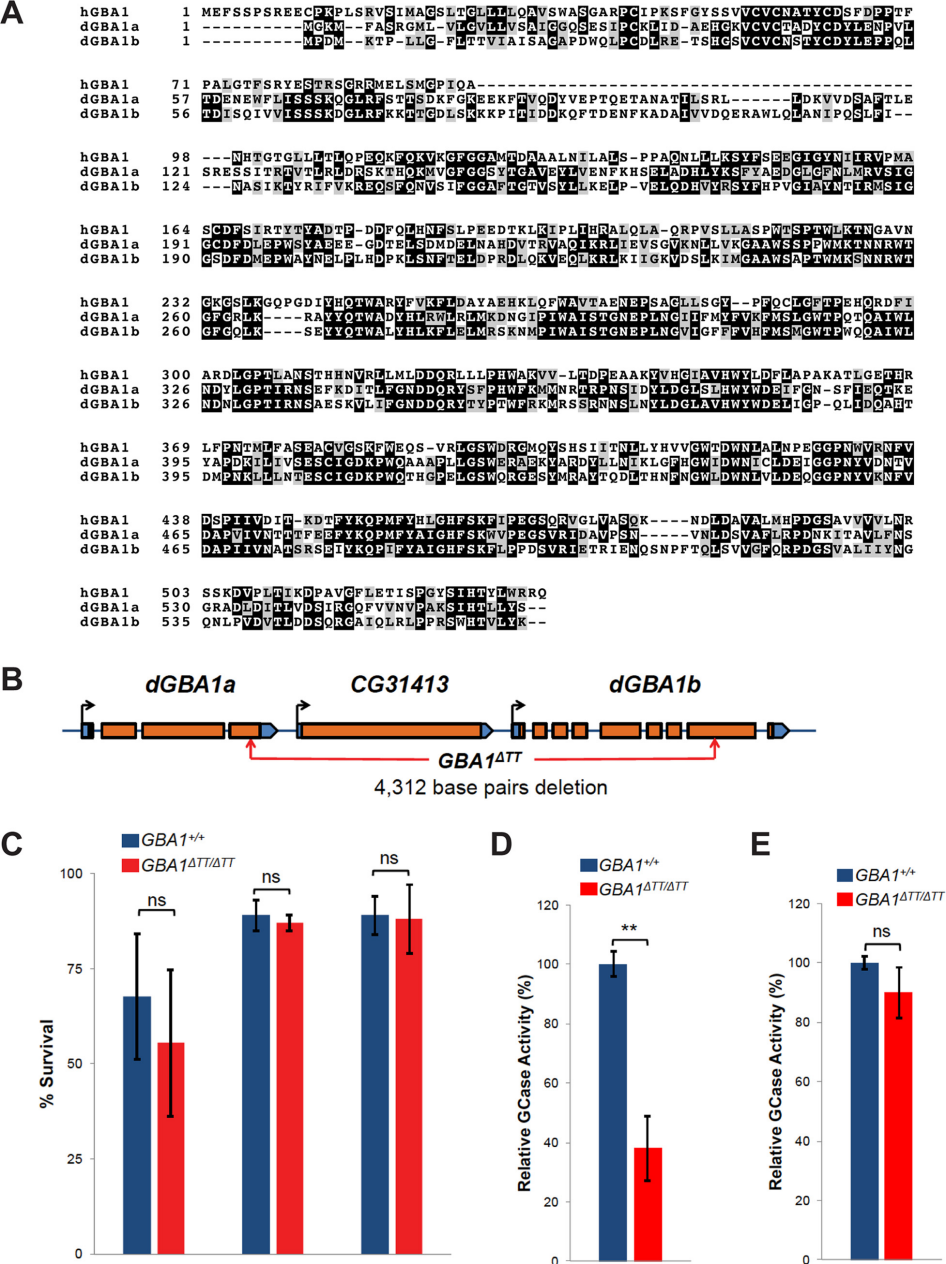
A *Drosophila dGBA1b* deletion results in glucocerebrosidase deficiency. (A) Comparison of protein sequence of human GBA1 and *Drosophila* dGBA1a and dGBA1b. Gray indicates similar residues, whereas black shading indicates identical residues. (B) Genomic organization of the *Drosophila GBA1* homologs, *dGBA1a* and *dGBA1b*, and the intervening *CG31413* gene. Orange and blue boxes represent coding and non-coding sequences, respectively. Black arrows indicate direction of transcription. Red arrows designate the breakpoints of the *GBA1*^*ΔTT*^ deletion allele. (C) There was no significant difference in the percentage of *GBA1*^*ΔTT*^ homozygotes and WT controls (*GBA1*^*+/+*^) that survived the embryo to 1st instar larval transition, the 3^rd^ instar larval to pupal stage transition, or the pupal to adult stage transition. (D) Relative glucocerebrosidase (GCase) enzyme activity from isolated heads of 14-day-old controls and *GBA1*^*ΔTT*^ homozygotes. (E) Relative GCase enzyme activity from bodies excluding heads of 14-day-old controls and *GBA1*^*ΔTT*^ homozygotes. Error bars represent standard error of the mean (s.e.m.), ns indicates p>0.05, **p<0.005 by Student *t* test in all results shown in this figure.

To pursue a loss-of-function analysis of the *GBA1* gene, we conducted a BLAST search for *Drosophila* homologs using the human glucocerebrosidase protein as a query sequence. This search revealed two *Drosophila GBA1* homologs (*CG31148* and *CG31414*), each of which shares 32% amino acid identity (50% similarity) to human glucocerebrosidase (Fig 1A). In accordance with a recent study [16], we hereafter refer to the *CG31148* and *CG31414* genes as *dGBA1a* and *dGBA1b*, respectively. *dGBA1a* and *dGBA1b* reside approximately 2 kb apart on the right arm of chromosome 3 with a single gene (*CG31413*) situated between them (Fig 1B). Data from the *Drosophila* modENCODE [17] and FlyAtlas [18] gene expression studies indicate that *dGBA1a* is expressed primarily or exclusively in the midgut, whereas *dGBA1b* is broadly expressed throughout development in a wide range of tissues, including the larval and adult brain. The gene situated between *dGBA1a* and *dGBA1b* (*CG31413*) encodes a putative quiescin sulfhydryl oxidase that is expressed exclusively in the male accessory gland, an organ that secretes proteins into the seminal fluid. We confirmed the expression patterns described by the modENCODE [17] and FlyAtlas [18] projects for *dGBA1a*, *dGBA1b* and *CG31413* using quantitative RT-PCR (qPCR) (S1 and S2B Figs). Given the expression patterns of *dGBA1a* and *dGBA1b*, the *dGBA1b* gene appeared most relevant to our studies, and our work focused on this gene.

To explore the consequences of glucocerebrosidase deficiency in *Drosophila*, we created deletions of the *Drosophila GBA1* homologs using publicly available transposon insertions in *dGBA1a* and *dGBA1b* [18]. Among the >200 candidate deletions generated using this approach was a 4.3 kb deletion (designated *GBA1*^*ΔTT*^) that removes the first 433 amino-terminal codons of the *dGBA1b* gene and the carboxy-terminal 33 codons of the *dGBA1a* gene (Fig 1B). This deletion also removes *CG31413* that is situated between *dGBA1a* and *dGBA1b* (Fig 1B). In addition to the *GBA1*^*ΔTT*^ deletion, we also generated a precise excision of the transposon used to create the *GBA1*^*ΔTT*^ allele (a chromosome in which the transposon was excised, but did not produce a deletion), which was used as an isogenic wild type (WT) control in all of our studies (designated *GBA1*^*+/+*^). The *GBA1*^*ΔTT*^ allele results in the production of a hybrid transcript fusing the amino terminal *dGBA1a* coding sequence to the carboxy terminal *dGBA1b* sequences (Figs 1B, S2B and S2C) but does not create an in-frame fusion of the *dGBA1a* and *dGBA1b* coding sequences, and therefore would not be expected to yield a chimeric glucocerebrosidase protein. Given that the *GBA1*^*ΔTT*^ deletion eliminates the presumptive promoter and >3/4 of the amino terminal coding sequence of *dGBA1b*, resulting in ~75% reduced expression of a *GBA1a* transcript lacking the final 33 codons of *dGBA1a*, we conclude that it likely represents a null allele of *dGBA1b* and a hypomorphic allele of *dGBA1a*. The residual expression of this fusion transcript was nearly undetectable in heads from *GBA1*^*ΔTT*^ homozygotes (S2C Fig).

*GBA1*^*ΔTT*^ homozygotes were viable, fertile and had no detectable morphological abnormalities (Fig 1C). However, before proceeding with a full characterization of *GBA1*^*ΔTT*^ phenotypes, we first sought to confirm that this deletion alters glucocerebrosidase activity and to test whether inactivation of the *CG31413* gene is likely to contribute to the *GBA1*^*ΔTT*^ phenotypes. We compared glucocerebrosidase activity in 14-day-old control flies and *GBA1*^*ΔTT*^ homozygotes using a standard fluorescent assay (see Materials and Methods). Since *dGBA1a* is expressed in the midgut and *dGBA1b* is expressed ubiquitously, we hypothesized that *dGBA1b* is responsible for most of the glucocerebrosidase activity in fly heads and should be absent or severely reduced in *GBA1*^*ΔTT*^ mutant heads. Consistent with our hypothesis glucocerebrosidase activity was reduced by ~60% in *GBA1*^*ΔTT*^ mutant heads compared to control fly bodies (Fig 1D) However, we did not observe a significant reduction in glucocerebrosidase activity in *GBA1*^*ΔTT*^ mutant bodies (Fig 1E), suggesting that most of the glucocerebrosidase activity in bodies derives from *dGBA1a* expression in the midgut, and that the function of *dGBA1a* is retained in *GBA1*^*ΔTT*^ homozygotes.

Although the *CG31413* gene is only expressed in the male accessory gland, implying that mutations in this gene should only influence male fertility, we obtained and tested a stock bearing a transposon insertion within the single exon of *CG31413*, which is predicted to interrupt the coding sequence of this 561-amino-acid protein at amino acid position 119 (S4A Fig). qPCR revealed a reduction in the expression of CG31413 in flies homozygous for this transposon insertion by ~90% compared to controls (S4C Fig). This mutation *in trans* to *GBA1*^*ΔTT*^ did not detectably influence lifespan or behavior (S4D–S4F Fig). Thus, our data does not support involvement of *CG31413* in the phenotypes of *GBA1*^*ΔTT*^ homozygotes.

**Fig 2:**
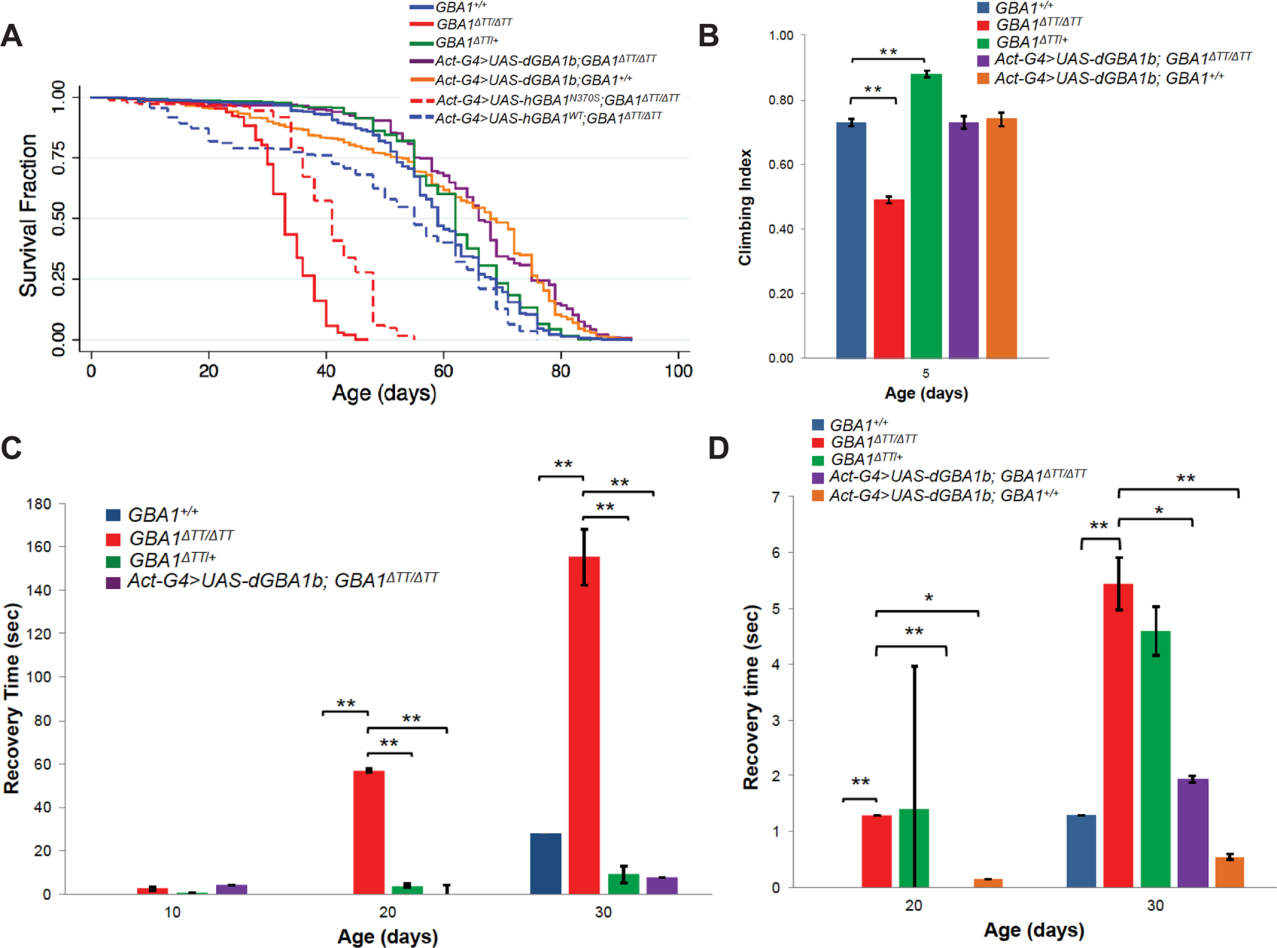
*GBA1*^*ΔTT*^ homozygotes exhibit shortened lifespan and behavioral phenotypes consistent with neuronal dysfunction. (A) Kaplan-Meier survival curves of WT controls (*GBA1*^*+/+*^), *GBA1*^*ΔTT*^ heterozygotes (*GBA1*^*ΔTT/+*^), *GBA1*^*ΔTT*^ homozygotes (*GBA1*^*ΔTT/ΔTT*^), WT controls ectopically expressing *Drosophila* WT *dGBA1b* using the *Actin GAL4* driver (*Actin-GAL4>UAS-GBA1b;GBA*^*+/+*^), *GBA1*^*ΔTT*^ homozygotes ectopically expressing *Drosophila* WT *dGBA1b* using the *Actin GAL4* driver *(Act-GAL4>UAS-GBA1b;GBA1*^*ΔTT/ΔTT*^), *GBA1*^*ΔTT*^ homozygotes ectopically expressing human WT *GBA1* using the *Actin GAL4* driver (*Act-GAL4>UAS-hGBA1*^*WT*^*;GBA1*^*ΔTT/ΔTT*^), and *GBA1*^*ΔTT*^ homozygotes ectopically expressing human *GBA1* harboring the p.*N370S* mutation using the *Actin GAL4* driver (*Act-GAL4>UAS-hGBA1*^*N370S*^*;GBA1*^*ΔTT/ΔTT*^). (B) Climbing index of 5-day-old flies of indicated genotypes as described in A. (C) Recovery time from mechanical stress (bang sensitivity) of flies of given genotypes as described in A at given adult ages. (D) Recovery time from heat stress of flies of given phenotypes as described in A at given adult ages. Error bars represent s.e.m., *p<0.05, **p<0.005 by Student *t* test in all results shown in this figure.

Although *GBA1*^*ΔTT*^ heterozygotes and homozygotes were viable and fertile, *GBA1*^*ΔTT*^ homozygotes were significantly shorter-lived than WT controls and *GBA1*^*ΔTT*^ heterozygotes, with mean survival of 33 days in *GBA1*^*ΔTT*^ homozygotes compared to mean survival of 59 days in controls and 62 days in *GBA1*^*ΔTT*^ heterozygotes (Fig 2A, S1 Table). *GBA1*^*ΔTT*^ homozygotes also exhibited a variety of age-dependent behavioral abnormalities, including a progressive climbing defect that was present as early as 5 days of age (Fig 2B; S3E Fig), delayed recovery from heat-induced paralysis (Fig 2C), and delayed recovery from temporary paralysis after mechanical stress (bang sensitivity) (Fig 2D). *GBA1*^*ΔTT*^ heterozygotes also exhibited a mild bang sensitivity phenotype at advanced ages, but did not exhibit phenotypes in the other behavioral assays performed (Fig 2A–2D).

Ectopic expression of *dGBA1b* using the *Actin*-*GAL4* driver ameliorated the decreased lifespan, climbing defect, heat and bang sensitivity phenotypes of *GBA1*^*ΔTT*^ homozygotes (Fig 2A–2D, S1 Table). Ectopic expression of human WT *GBA1* also rescued the lifespan defect of *GBA1*^*ΔTT*^ homozygotes to a greater extent than did expression of a human *GBA1* construct encoding the p.*N370S* missense allele (S4 Fig), one of the most common pathogenic mutations causing GD (Fig 2A, S1 Table). Together, these findings suggest that the phenotypes of *GBA1*^*ΔTT*^ homozygotes result from loss of *dGBA1b* function, and that *Drosophila dGBA1b* is functionally equivalent to human *GBA1*.

### Glucocerebrosidase deficiency results in a memory defect and neurodegeneration, but no loss of dopaminergic neurons

**Fig 3:**
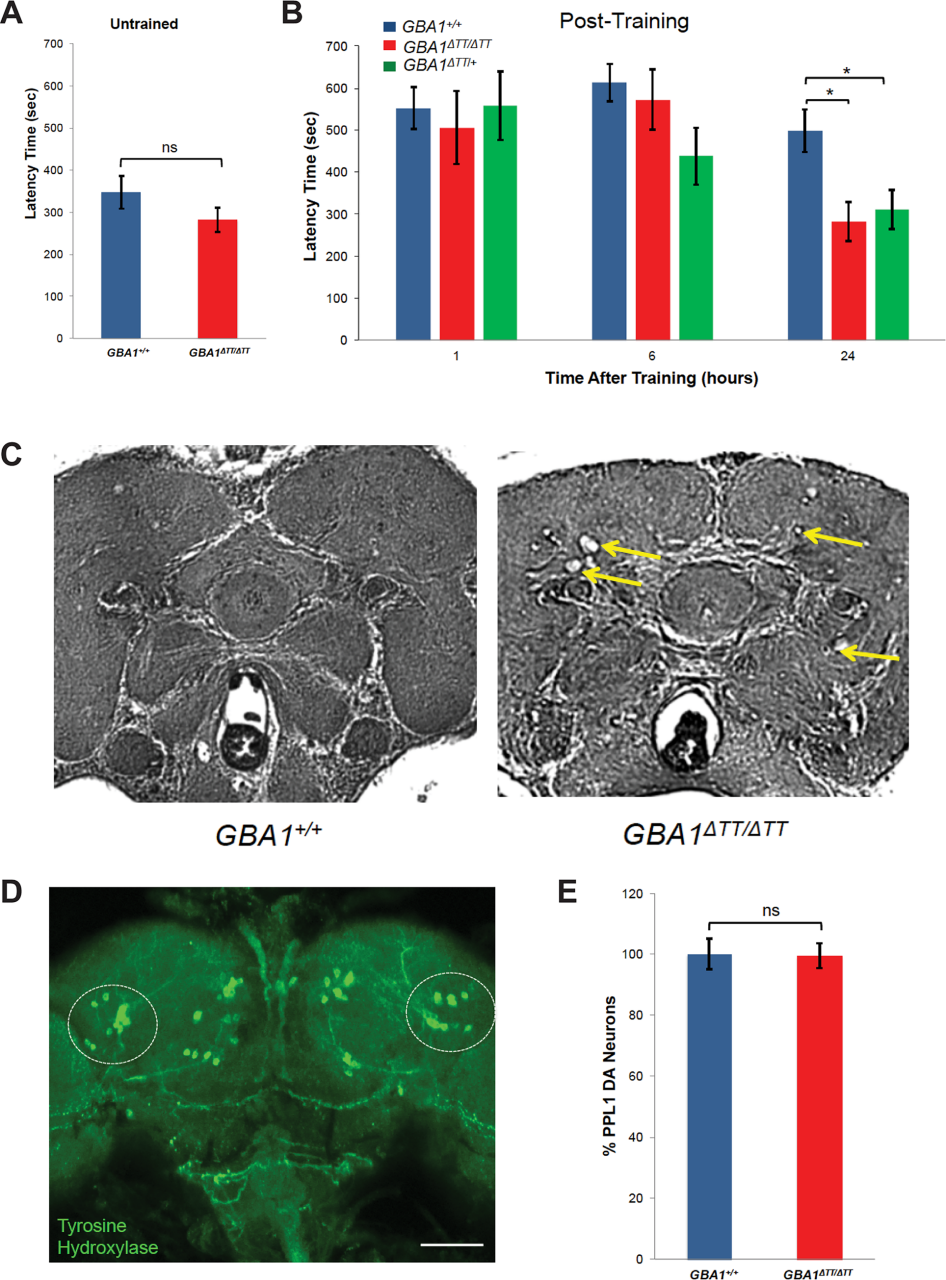
*GBA1*^*ΔTT*^ homozygotes display a memory deficit and neurodegeneration, but do not have dopaminergic neuron loss. (A) Latency time to initiate courtship in untrained 14-day-old males of indicated genotype. (B) Latency time to initiate courtship in 14-day-old males of indicated genotypes at 1 hour, 6 hours and 24 hours following training using a conditioned mating assay. Note the longer latency times in trained males (B) relative to untrained males (A). (C) Representative paraffin-embedded H&E-stained brain sections from 30-day-old controls and *GBA1*^*ΔTT*^ homozygotes. Yellow arrows indicate vacuoles. (D) Representative image of a projected Z-series of a control adult *Drosophila* brain stained with anti-Tyrosine Hydroxylase to label dopaminergic (DA) neurons. DA neurons within the PPL1 cluster are indicated by the circled regions. Scale bar, 200 μm. (E) Relative number of DA neurons within the PPL1 cluster of 30-day-old *GBA1*^*ΔTT*^ homozygotes (*GBA1*^*ΔTT/ΔTT*^) *N* = 19, normalized to age-matched WT controls (*GBA1*^*+/+*^) *N* = 21. There was no significant difference between the number of DA neurons within the PPL1 cluster per genotype by Student *t* test. Error bars represent s.e.m., ns indicates *p*>0.05, *p<0.05, **p<0.005 by Student *t* test in all results shown in this figure.

Because *GBA1* mutations are frequently associated with cognitive impairment [3, 4], and the phenotypes of the *GBA1*^*ΔTT*^ homozygotes are similar to those of previously characterized *Drosophila* models of neurological dysfunction [19, 20], we tested whether *GBA1*^*ΔTT*^ homozygotes also exhibited a cognitive defect. To perform this analysis, we used a simple courtship assay of learning and memory. After mating, *Drosophila* females will not mate again for at least 24 hours, and during this time will vigorously reject mating advances from males. Virgin males placed with recently mated females learn that their advances are likely to be rejected, and demonstrate this learned behavior with an increased latency to initiate courtship. The latency to initiate courtship was similar in 14-day-old untrained *GBA1*^*ΔTT*^ homozygotes and controls (Fig 3A), indicating that *GBA1*^*ΔTT*^ homozygotes do not have a motor impairment that adversely influences courtship. The *GBA1*^*ΔTT*^ homozygotes also displayed normal learning, as the increase in latency to initiate courtship 1 hour following training was the same in both *GBA1*^*ΔTT*^ homozygotes and controls (Fig 3B). However, while control flies still showed increased latency to initiate courtship 24 hours after training, courtship latency in *GBA1*^*ΔTT*^ homozygotes and heterozygotes had returned to the level seen in untrained animals (Fig 3B). These findings indicate that *GBA1*^*ΔTT*^ heterozygotes and homozygotes are capable of remembering for short, but not long, periods of time.

To test whether the phenotypes of *GBA1*^*ΔTT*^ homozygotes are caused by neurodegeneration, we examined brain sections from paraffin-embedded fly heads for gross neuropathology. We found that 30-day-old *GBA1*^*ΔTT*^ homozygotes exhibited substantially increased brain vacuolization relative to age-matched controls (Fig 3C). Similar vacuoles have been characterized in other *Drosophila* neurodegenerative models, including *Drosophila* models of tauopathy [21], Alzheimer’s disease [22], and amyotrophic lateral sclerosis [23], indicating that *GBA1*^*ΔTT*^ homozygotes have a neurodegenerative phenotype. As there is dopaminergic neuron loss in PD and DLB, and prior work demonstrated significant dopaminergic neuron loss in the *protocerebral posterior lateral 1* (PPL1) cluster of dopaminergic neurons (outlined in Fig 3D) in several *Drosophila* models of PD, including *parkin* mutants [24] and α-synuclein transgenic flies [25], we tested whether *GBA1*^*ΔTT*^ homozygotes also exhibit dopaminergic neuron loss. Despite the increased presence of vacuoles in 30-day-old *GBA1*^*ΔTT*^ homozygote brains compared to age-matched controls (Fig 3C), there was no significant loss of dopaminergic neurons in the PPL1 cluster in 30-day-old *GBA1*^*ΔTT*^ homozygotes (Fig 3E).

### Glucocerebrosidase deficiency leads to the accumulation of protein aggregates that are normally degraded through autophagy

**Fig 4:**
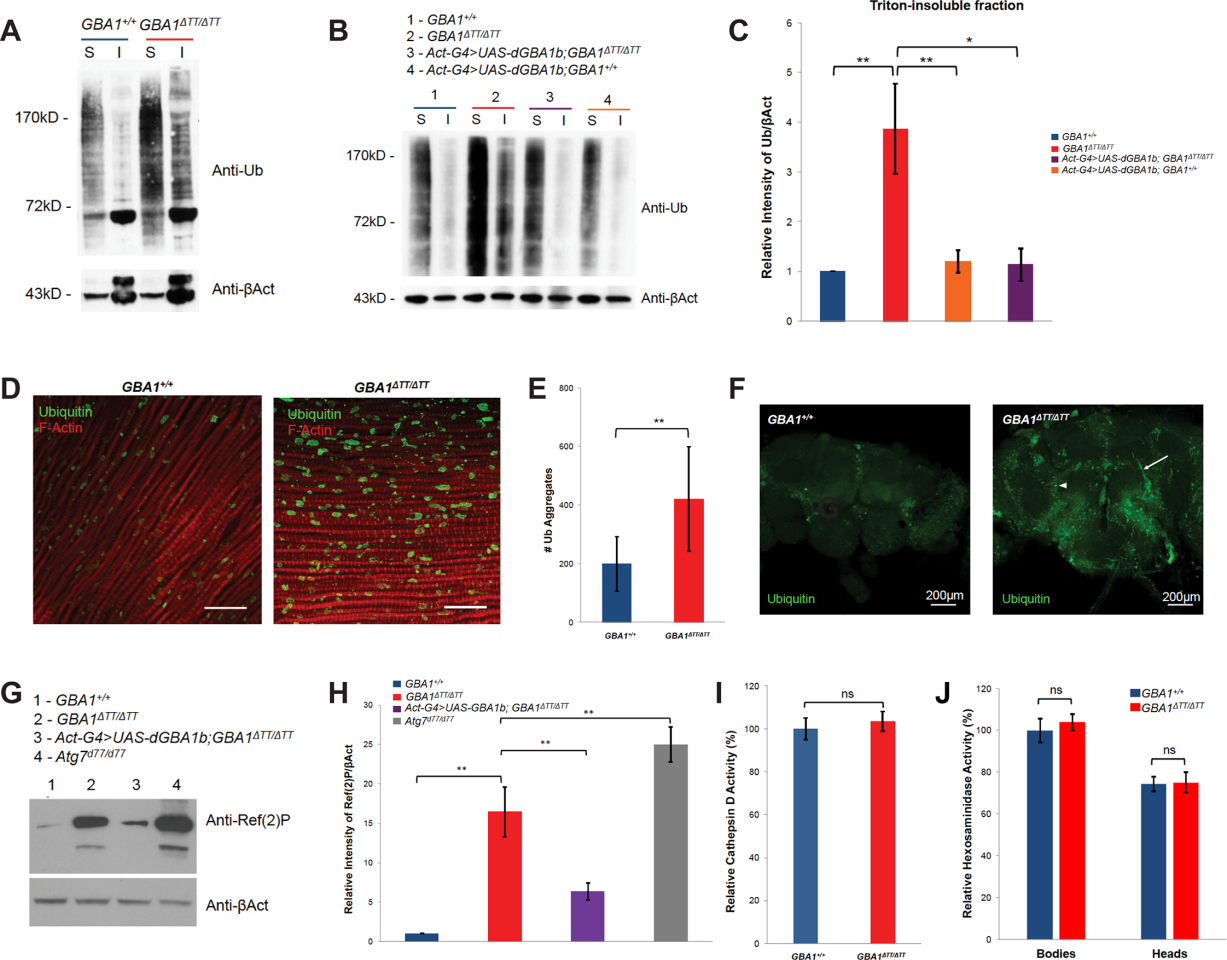
*GBA1*^*ΔTT/ΔTT*^ exhibit increased accumulation of soluble and insoluble ubiquitinated proteins. (A) Western blot using antiserum to ubiquitin (Ub) of Triton-soluble (S) and Triton–insoluble (I) protein fractions from whole 30-day-old WT controls (*GBA1*^*+/+*^) and *GBA1*^*ΔTT*^ homozygotes (*GBA1*^*ΔTT/ΔTT*^), using anti-β-Actin (βAct) as a loading control. The intense ubiquitin-positive band immediately below the 72kD marker and the upper βAct band likely represents arthirin, a 55kD mono-ubiquitinated form of β-Actin that is present exclusively in the indirect flight muscle [68]. (B) Western blot probed for Ub of Triton–soluble (S) and Triton–insoluble (I) protein fractions from heads of flies of 30-day-old *GBA1*^*+/+*^, *GBA1*^*ΔTT/ΔTT*^, WT controls ectopically expressing *Drosophila* WT *dGBA1b* using the *Actin GAL4* driver (*Actin-GAL4>UAS-GBA1b;GBA*^*+/+*^), and *GBA1*^*ΔTT*^ homozygotes ectopically expressing *Drosophila* WT *dGBA1b* using the *Actin GAL4* driver *(Act-GAL4>UAS-GBAb;GBA1*^*ΔTT/ΔTT*^), using βAct as a loading control. (C) Densitometric quantification of Ub signal in the Triton-insoluble fraction from the heads of flies of the indicated genotypes. Levels of Ub signal per genotype were normalized to respective βAct loading controls, and these ratios were in turn normalized to the insoluble Ub level of *GBA1*^*+/+*^. (D) Representative immunofluorescent staining of thoracic muscle from 30-day-old *GBA1*^*+/+*^ and *GBA1*^*ΔTT/ΔTT*^ flies with anti-Ub and anti-F-Actin. Scale bars, 20 μm. (E) Quantification of Ub-positive objects within 10-μm-thick *Z*-stacks of thoracic muscle of 30-day-old *GBA1*^*+/+*^ and *GBA1*^*ΔTT/ΔTT*^ flies. At least 9 separate Z-stacks were analyzed per genotype. Error bars represent standard deviation. (F) Representative anti-Ub immunofluorescent staining of 30-day-old whole brains from *GBA1*^*+/+*^ and *GBA1*^*ΔTT/ΔTT*^ flies. Scale bar, 200 μm. Arrowhead indicates punctate staining pattern, arrow indicates filamentous staining pattern. (G) Western blot using antiserum to Ref(2)P of whole 10-day-old flies of the indicated genotypes, including *Atg7* null flies *(Atg7*^*d77/d77*^). βAct was used as a loading control. (H) Densitometric quantification of Ref(2)P signal from 10-day-old whole flies of the indicated genotypes. Levels of Ref(2)P signal per genotype were normalized to respective βAct loading controls, and these ratios were in turn normalized to the Ref(2)p level of *GBA1*^*+/+*^. (I) Cathepsin D activity in whole 7-day-old *GBA1*^*ΔTT*^ homozygotes relative to age-matched controls. (J) Hexosaminidase activity in bodies excluding heads, and isolated heads of 7-day-old *GBA1*^*ΔTT*^ homozygotes relative to age-matched controls. Error bars represent s.e.m. unless indicated, ns indicates *p*>0.05, *p<0.05, **p<0.005 by Student *t* test in all results shown in this figure.

Previous work has shown that insoluble ubiquitinated protein aggregates accumulate naturally with age in *Drosophila*, and that genetic perturbations of autophagy factors influence the formation of these aggregates, suggesting that they are normally degraded in the lysosome [26–28]. We postulated that these insoluble ubiquitinated protein aggregates would be more abundant in *GBA1*^*ΔTT*^ homozygotes if glucocerebrosidase deficiency impairs lysosomal protein degradation. Consistent with our hypothesis, we found that insoluble ubiquitinated protein aggregates were far more abundant in *GBA1*^*ΔTT*^ homozygotes in protein extracts from whole flies (Fig 4A) as well as isolated fly heads (Fig 4B and 4C). Soluble ubiquitinated proteins were also more abundant in *GBA1*^*ΔTT*^ homozygotes, suggesting that their ability to degrade ubiquitinated proteins is generally impaired (Fig 4A and 4B). These effects were reversed upon ectopic expression of *dGBA1b*, confirming that this phenotype is a consequence of *dGBA1b* deficiency (Fig 4B and 4C). Ubiquitinated aggregates were also visualized in fixed thoracic muscle (Fig 4D and 4E) and whole brains (Fig 4F), with increased aggregate formation seen in *GBA1*^*ΔTT*^ homozygotes compared to age-matched controls. Interestingly, *GBA1*^*ΔTT*^ homozygote brains included both punctate (arrowhead) and filamentous (arrow) staining patterns suggestive of aggregates within both cell bodies and cell projections, whereas only the punctate staining pattern was observed in age-matched controls.

To determine whether increased ubiquitinated protein aggregation in *GBA1*^*ΔTT*^ homozygotes is due to impaired lysosomal protein degradation, we examined whether Ref(2)P, the *Drosophila* homolog of *SQSTM1*/p62, is more abundant in *GBA1*^*ΔTT*^ homozygotes compared to WT controls. *SQSTM1*/p62 is important for selective targeting of ubiquitinated proteins for lysosomal degradation and accumulates when autophagic flux is impaired [29, 30]. Similar to the autophagy mutant, *Atg7*, we found a dramatic increase in Ref(2)P in *GBA1*^*ΔTT*^ homozygotes compared to age-matched controls and *GBA1*^*ΔTT*^ homozygotes ectopically expressing *dGBA1b*, (Fig 4G and 4H). These findings support the model that glucocerebrosidase deficiency impairs the lysosomal degradation of ubiquitinated protein aggregates, and that the phenotypes of *GBA1*^*ΔTT*^ homozygotes are a consequence of defective flux of autophagic vesicles. However, there was no significant difference in Cathepsin D or hexosaminidase enzyme activity, other lysosomal enzymes, between controls and *GBA1*^*ΔTT*^ mutants (Fig 4I and 4J), suggesting that glucocerebrosidase deficiency does not generally impair lysosome enzymatic function.

### Glucocerebrosidase deficiency enhances α-synuclein aggregation, but has little effect on α-synuclein toxicity

α-synuclein protein aggregation and Lewy body formation are hallmark pathologic characteristics of *GBA1*-associated neurodegenerative diseases [31]. Moreover, duplications or triplications causing an overabundance of α-synuclein protein cause heritable forms of PD [32] and α-synuclein protein is at least partially degraded in the lysosome [33]. Recent work in mammalian models, cell culture and post-mortem tissue from individuals with PD indicates that a reciprocal relationship exists between glucocerebrosidase deficiency and the abundance of α-synuclein aggregates [14, 31, 34, 35]. Together, these findings raise the possibility that *GBA1* mutations act by influencing the abundance of toxic α-synuclein aggregates. However, it is unclear from previous work how much α-synuclein contributes to the pathogenesis of disorders caused by *GBA1* mutations [14, 31]. Our *Drosophila* model of glucocerebrosidase deficiency provides a unique opportunity to address this issue, as there is no *Drosophila* ortholog to human α-synuclein.

**Fig 5:**
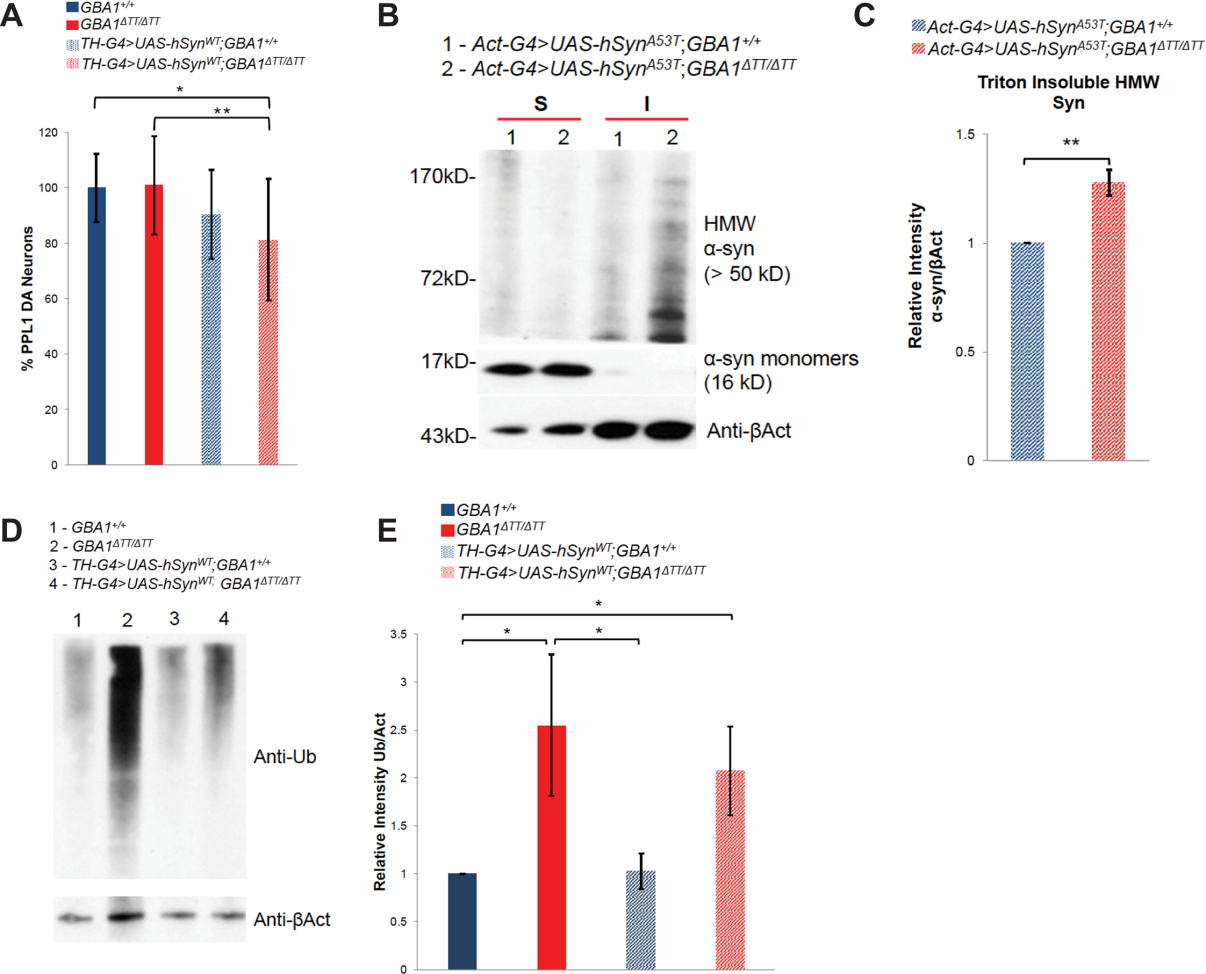
Glucocerebrosidase deficiency mildly enhances α-synuclein toxicity in neurons and increases the abundance of α-synuclein aggregates. (A) Relative number of DA neurons within the PPL1 cluster of 30-day-old flies, normalized to WT controls. WT controls (*GBA1*^*+/+*^) *N* = 12, *GBA1*^*ΔTT*^ homozygotes (*GBA1*^*ΔTT/ΔTT*^) *N* = 22, WT controls expressing α-synuclein in DA neurons (*TH-G4>UAS-syn*^*WT*^;*GBA1*^*+/+*^) *N* = 16, and *GBA1*^*ΔTT*^ homozygotes expressing α-synuclein in DA neurons (*TH-G4>UAS-syn*^*WT*^;*GBA1*^*ΔTT/ΔTT*^) *N* = 28. (B) Western blot analysis using antiserum to α-synuclein (α-syn) of Triton-insoluble protein fractions from heads of 10-day-old WT controls (*GBA1*^*+/+*^), *GBA1*^*ΔTT*^ homozygotes (*GBA1*^*ΔTT/ΔTT*^), WT controls ubiquitously expressing α-synuclein with the p.A53T mutation (*Act-G4>UAS-syn*^*A53T*^;*GBA1*^*+/+*^), and *GBA1*^*ΔTT*^ homozygotes ubiquitously expressing α-synuclein with the p.A53T mutation (*Act-G4>UAS-syn*^*A53T*^;*GBA1*^*ΔTT/ΔTT*^). βAct was used as a loading control. (C) Densitometric quantification of α-syn signal in the Triton-insoluble fraction from 10-day-old heads of flies of indicated genotypes as described in B. The level of HMW α-syn signal in the Triton-insoluble fraction in *GBA1*^*ΔTT/ΔTT*^ was normalized to its βAct loading control, and this ratio was in turn normalized to the insoluble α-syn/βAct ratio of *GBA1*^*+/+*^, *N =* 3. (D) Western blot analysis using an antiserum to Ubiquitin (anti-Ub) of Triton–soluble (S) and–insoluble (I) protein extracts from heads of controls ectopically expressing WT α-synuclein using the *TH-GAL4* driver (*TH-G4>UAS-syn*^*WT*^*;GBA1*^*+/+*^) and *GBA1*^*ΔTT*^ homozygotes ectopically expressing WT α-synuclein using the *TH-GAL4* driver (*TH-G4>UAS-syn*^*WT*^*;GBA1*^*ΔTT/ΔTT*^). βAct was used as a loading control. (E) Densitometric quantification of anti-Ub levels in Triton-insoluble protein fractions from heads of flies of indicated genotypes as described in D. Levels of Ub signal per genotype were normalized to respective βAct loading controls, and the ratio of Ub/βAct in *Th-G4>UAS-syn*^*WT*^;*GBA1*^*ΔTT/ΔTT*^ was normalized to the ratio of α-syn/βAct in *TH-G4>UAS-syn*^*WT*^;*GBA1*^*+/+*^. Error bars represent s.e.m. unless indicated, *p<0.05, **p<0.005 by Student *t* test in all results shown in this figure.

**Fig 6:**
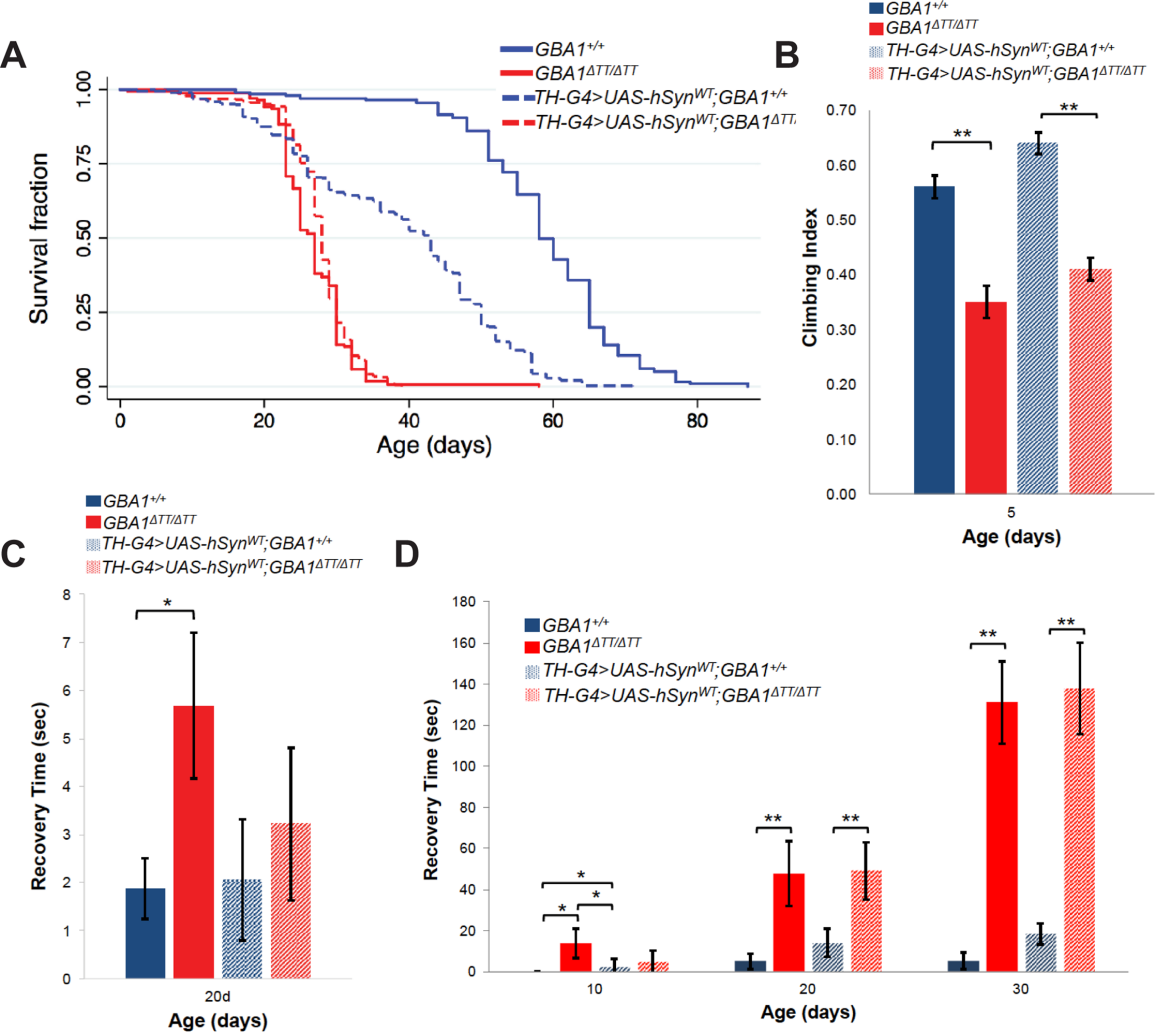
α-synuclein expression does not enhance *dGBA1b* deficient fly phenotypes. (A) Kaplan-Meier survival curves of lifespans of WT controls, *GBA1*^*ΔTT*^ homozygotes, WT controls expressing α-synuclein in dopaminergic neurons (*TH-G4>UAS-syn*^*WT*^;*GBA1*^*+/+*^), and *GBA1*^*ΔTT*^ homozygotes expressing α-synucleinin dopaminergic neurons (*TH-G4>UAS-syn*^*WT*^;*GBA1*^*ΔTT/ΔTT*^). (B) Climbing index of 5-day-old flies of given genotypes. (C) Recovery time from temporary paralysis due to mechanical stress (bang sensitivity) of flies of given phenotypes at given adult age. (D) Recovery time from heat stress of flies of given genotypes at given adult ages. Error bars represent s.e.m., *p<0.05, **p<0.005 by Student *t* test in all results shown in this figure.

To test whether the lack of dopaminergic neuron loss in *GBA1*^*ΔTT*^ homozygotes (Fig 3E) is explained by the absence of a *Drosophila* ortholog to human α-synuclein, we ectopically expressed human α-synuclein in a *GBA1*^*ΔTT*^ homozygous background. In previous work we showed that abundant expression of human α-synuclein using two copies of the *Tyrosine Hydroxylase (TH) GAL4* driver, and two copies of a *UAS-α-synuclein* transgene resulted in mild degeneration of dopaminergic neurons [25]. Using this identical α-synuclein expression paradigm, we observed a trend towards mild loss of dopaminergic neurons in the PPL1 cluster of WT controls that did not reach statistical significance (Fig 5A). This trend was further enhanced and reached statistical significance in *GBA1*^*ΔTT*^ homozygotes ectopically expressing α-synuclein (Fig 5A), indicating that glucocerebrosidase deficiency mildly enhanced α-synuclein toxicity in dopaminergic neurons. However, expression of α-synuclein in dopaminergic neurons did not significantly enhance the shortened lifespan, climbing deficits, sensitivity to mechanical stress or heat stress of *GBA1*^*ΔTT*^ mutants (Fig 6A–6D).

To test the hypothesis that glucocerebrosidase deficiency impairs lysosomal degradation of α-synuclein protein and leads to the accumulation of toxic α-synuclein aggregates, we compared the abundance of *α-synuclein* aggregates in *GBA1*^*ΔTT*^ homozygotes and controls. We were unable to detect aggregation of WT α-synuclein by Western blot analysis. However, expression of an α-synuclein variant harboring the pathogenic missense mutation p.A53T (α-synuclein^A53T^), which causes autosomal dominant early-onset PD and accelerated insoluble fibril formation [36], resulted in the appearance of high molecular weight (HMW) α-synuclein species (>50kD) in the Triton-insoluble protein fraction (Fig 5B). We further found that the abundance of these HMW α-synuclein species were increased in *GBA1*^*ΔTT*^ homozygotes ubiquitously expressing α-synuclein^A53T^ relative to control flies expressing human α-synuclein^A53T^ (Fig 5B and 5C). These findings indicate that glucocerebrosidase deficiency impairs degradation of HMW α-synuclein aggregates, or promotes the kinetics of formation of these aggregates. However, ubiquitously expressing α-synuclein^A53T^ in *GBA1*^*ΔTT*^ homozygotes had no significant influence on the phenotypes of the *GBA1*^*ΔTT*^ homozygotes (S5C and S5D Fig) indicating that the enhanced α-synuclein aggregate formation conferred by glucocerebrosidase deficiency was not matched by a detectable increase in α-synuclein toxicity.

Since α-synuclein is a major component of Lewy bodies and recent work suggests that α-synuclein fibrils may contribute to PD pathogenesis in a non-cell-autonomous fashion by seeding protein aggregation in a prion-like mechanism [37–39], we hypothesized that ectopically expressed α-synuclein might increase the pool of insoluble ubiquitinated proteins seen in *GBA1*^*ΔTT*^ homozygotes. Surprisingly, there was no significant enhancement of total insoluble ubiquitinated protein levels with ectopic expression of either WT α-synuclein or the A53T variant of α-synuclein in either WT controls or *GBA1*^*ΔTT*^ homozygotes relative to controls and *GBA1*^*ΔTT*^ homozygotes without α-synuclein expression (Fig 5D and 5E; S5A and S5B Fig). These results indicate that α-synuclein expression does not independently or synergistically influence the increased protein aggregation resulting from *dGBA1b* deficiency, even when expressing a mutant form of α-synuclein prone to forming fibrils.

## Discussion

To gain insight into the molecular mechanisms underlying PD, DLB and neuronopathic forms of GD, we developed a *Drosophila* model of glucocerebrosidase deficiency. Glucocerebrosidase deficiency in *Drosophila* results in shortened lifespan, a variety of age-dependent behavioral phenotypes, neurodegeneration and the accumulation of insoluble proteins that are normally degraded through an autophagic mechanism. While these phenotypes are reminiscent of α-synucleinopathies [40, 41], glucocerebrosidase deficiency only mildly influenced the neuronal toxicity and aggregation of α-synuclein, and ectopic expression of α-synuclein did not significantly enhance the glucocerebrosidase deficient phenotypes. Together, our findings indicate that the pathological consequences of glucocerebrosidase deficiency in *Drosophila* are largely independent of α-synuclein, and that glucocerebrosidase deficiency is the major contributor to pathology in diseases associated with *GBA1* mutations.

Our finding that α-synuclein is not a central participant in the pathogenesis associated with glucocerebrosidase deficiency is consistent with recent studies in two different fish species [42, 43]. The inverse correlation between glucocerebrosidase activity and α-synuclein aggregation in *Drosophila* is also consistent with previous studies in rodent models, vertebrate cell culture, post-mortem brain tissues from PD patients, and a recent study in *Drosophila* [14, 31, 34, 35]. Although we were only able to observe an influence of glucocerebrosidase deficiency on the aggregation of the p.A53T variant of α-synuclein, this finding may simply reflect the fact that this variant is more aggregation-prone, thus allowing us increased sensitivity to detect aggregation. However, our work showing the *dGBA1b* gene is the predominant *Drosophila GBA1* homolog expressed in the fly head contrasts with a recent report showing that neuronal inactivation of the *Drosophila dGBA1a* gene exacerbated the toxicity of α-synuclein in dopaminergic neurons and in the fly eye [16]. Additional studies will be required to fully address the role of *dGBA1a*, which appears to remain largely functional in our mutant, and to definitively rule out a role for the *CG31413* gene situated between *dGBA1a* and *dGBA1b* on the phenotypes of *GBA1*^*ΔTT*^ homozygotes.

Although our work indicates that glucocerebrosidase deficiency has little influence on the toxicity of α-synuclein, the association of *GBA1* mutations with PD and DLB frequently involves heterozygous carriers of *GBA1* missense alleles. This finding has led to the suggestion that the *GBA1* mutations act through a dominant toxic gain-of-function mechanism to cause PD and DLB, perhaps by seeding α-synuclein aggregates via a prion-like mechanism [37–39, 44]. Because our work involved a putative null allele of *dGBA1b*, we were unable to address this potential model of pathogenesis. Previous work also indicates that ectopic expression of human α-synuclein in *Drosophila* confers only mild phenotypic consequences [25, 45], so it is also possible that the influence of glucocerebrosidase deficiency on α-synuclein toxicity is not readily evident in *Drosophila*. While we fully acknowledge these potential confounds, several compelling observations suggest that a loss-of-function mechanism best explains the influence of *GBA1* mutations on PD and DLB. For example, many different *GBA1* mutations are associated with α-synucleinopathies, including putative null alleles [46, 47], and the molecular severity of a *GBA1* allele correlates with the risk of developing an α-synucleinopathy in heterozygous carriers [48]. Perhaps the strongest evidence for a loss-of-function mechanism is the finding that individuals with biallelic *GBA1* mutations have a substantially elevated risk for developing PD relative to heterozygous *GBA1* mutation carriers [3, 4, 49]. Together these findings offer support for the relevance of animal models bearing null alleles of the *GBA1* gene [16, 42, 43], including our fly model of glucocerebrosidase deficiency, on our understanding of the influence of *GBA1* mutations in PD and DLB.

Our glucocerebrosidase deficient fly model should be a valuable tool in future work aimed at understanding the mechanisms underlying the neurodegenerative diseases associated with mutations in *GBA1*. Although glucocerebrosidase deficiency does not result in dopaminergic neuron degeneration in *Drosophila*, this finding does not necessarily challenge the utility of our fly model to understand the role of glucocerebrosidase deficiency in PD. Previous work has established that mutational inactivation of *Drosophila* homologs of genes involved in heritable forms of PD often results in phenotypes that appear discordant with those seen in humans [50–53]. For example, null mutations of the *PINK1* or *parkin* genes in *Drosophila* result in dramatic muscle degeneration and germ line defects that are not evident in humans bearing null mutations in these genes [52, 54]. However, substantial insight into the roles of PINK1 and Parkin in mitochondrial quality control derived directly from studies of PINK1 and Parkin in the *Drosophila* musculature and germ line [55]. We anticipate that similarly important insight into the mechanisms underlying neuronopathic GD, PD and DLB will come from studies of the phenotypes of our fly model of glucocerebrosidase deficiency.

Our work suggests at least two general mechanisms by which glucocerebrosidase deficiency triggers neuropathology. First, glucocerebrosidase deficiency may impair autophagy, resulting in increased protein aggregation. Our work in *GBA1b* mutant flies showing accumulation of Ref(2)P, HMW α-synuclein aggregates, and protein aggregates that are normally degraded through an autophagic mechanism supports this model. Glucocerebrosidase is an important lysosomal enzyme in lipid metabolism [56–58], and a deficiency in this enzyme could influence lysosome membrane fluidity, vesicular dynamics, and the biogenesis of lysosomes [57, 59, 60]. These effects could impair the trafficking of misfolded proteins to the lysosome and/or fusion of autophagic vacuoles. As we did not observe a decrease in Cathepsin D activity in *GBA1b* mutant flies, lysosomal function may not be impaired by glucocerebrosidase deficiency. Alternatively, glucocerebrosidase deficiency may promote the formation of protein aggregates, rather than impair their degradation. Lipid composition has been shown to influence the kinetics of formation of protein aggregates and α-synuclein fibrilization [61, 62], suggesting that an alteration in lipid composition resulting from glucocerebrosidase deficiency could accelerate the accumulation of protein aggregates. These aggregates might subsequently seed further aggregation in a prion-like mechanism. In support of this model, lipid composition has been shown to affect the kinetics of amyloid-β aggregation [63], and recent studies suggest that non-autonomous spreading of α-synuclein fibrils may contribute to PD pathogenesis (39–41). While it remains unclear whether the increased protein aggregates that we observed in *GBA1b* mutant flies are due to impaired degradation or accelerated formation of misfolded proteins, α-synuclein expression did not enhance the abundance of protein aggregates, arguing against an additive influence of α-synuclein on protein aggregation metabolism. Future experiments will be required to distinguish between these models and reveal the underlying mechanism of *GBA1*-mediated accumulation of protein aggregates.

## Materials and Methods

### Drosophila strains and cultures

Fly stocks were maintained on standard cornmeal-molasses food at 25°C. The human *GBA1* wild type and p.*N370S* transgenes were created by Alex Whitworth (MRC Mitochondrial Biology Unit) and were targeted to the attP landing site of Bloomington stock 24482—M{3xP3-RFP.attP'}ZH-51C (with M{vas-int.Dm}ZH-2A). Full length human α-synuclein was cloned into *pUASt*. *pUAS-synuclein*^*WT*^ was introduced into the *Drosophila* genome by injection in the Pallanck lab using standard P-element-mediated transformation techniques [64]. The full length *dGBA1b* transcript was cloned into *pUASTattB* and targeted to the attP landing site of Bloomington stock 36304—y[1] v[1]; P{y[+t7.7] = CaryP}attP40, and Bloomington stock 8622—y[1] w[67c23]; P{y[+t7.7] = CaryP}attP2. *pUASTattB-dGBA1b* was introduced into the *Drosophila* genome by Duke University Model System Genomics. All other stocks were obtained from the Bloomington Stock Center except for the *TH-GAL4* driver [65]. The TH-GAL4 transgene was randomly transpositioned to the second chromosome in the Pallanck lab.

### Generation of *GBA1*^*ΔTT*^ and isogenic wild type *GBA1*^*+*^ alleles

Publicly available stocks with Minos transposon insertions in *dGBA1a* (Mi{ET1}[52]*CG31148*[MB02296]) and *dGBA1b* (Mi{ET1}[45]*CG31414*[MB03039]) were used in combination with a source of transposase to create deletions that include the coding sequences of *dGBA1a* and *dGBA1b*. The following primers spanning the Minos transposon insertions in *CG31414* and *CG31148* were used to screen over 200 different lines created by transposase-mediated mutagenesis:

CG31148

Forward ACACCGGAGCAGTGGAATAC

Reverse GTCGGAGCTTTTTGAACTCG

CG31414

Forward CGCAACAATTCGCTGAACTAC

Reverse TGTTTGGGTCAAAAACAGCA

Whole genome sequencing using 100-bp paired-end reads on the Illumina HiSeq2000 platform was used to map the exact breakpoints of the *GBA1*^*ΔTT*^ allele. Specifically, reads were aligned and processed against r5.43 of the *D*. *melanogaster* genome using BWA and Samtools. Breakpoints were confirmed by visual inspection using the Integrated Genome Viewer.

### Detection of possible fusion transcript due to *GBA1*^*ΔTT*^ allele

The following primers were used to amplify and quantitate by qPCR a possible fusion transcript from cDNA isolated from 20 whole *GBA1*^*ΔTT*^ homozygotes:

Primer 1: TCTCCAAGTGGGTTCCAGAG

Primer 2: TGAAGTTGTGCGTCAGATCC

Primer 3: GACCGGACAACAAAATCACC

Primer 4: CGGAGCATCCACAAAGTTCT

### Gene expression analysis

RNA was extracted from 20 whole bodies, 20 bodies without heads, or 60 heads from 5 day old male flies (unless otherwise indicated) of the indicated genotype, using TRIzol (Life Technologies) and Direct-zol RNA MiniPrep extraction kit (Zymo Research). cDNA was generated using the iSCRIPT cDNA Synthesis kit (Bio-Rad). Quantitative real-time PCR (qPCR)-based quantification was performed for the following genes using the indicated primers:

GBA1a

Forward ACGATGACCAACGCTATTCC

Reverse ATACCAGTGCAGCGATAGCC

dGBA1b

Forward GAACCAGAGCAATCCCTTCA

Reverse TCATCGAGAGTCACGTCCAC

CG31413

Forward CAAGTCCCTTGAAGCCAGAG

Reverse CGAAGGACGAGAGGCAATAC

Rap2L

Forward CCGCTGAAGGTAATGCCTTG

Reverse CGTTTATCCGATCCTTTGCAGA

qPCR was performed using iTaq Universal SYBR Green Supermix (Bio Rad) and a LightCycler 480 (Roche) machine. The delta-delta log_2_ method was used to calculate fold change in expression levels. *Rap2L* was used as the internal control, as the expression of this gene has been reported as the most invariant across different genotypes and ages [66]. Each experiment was performed at least three times.

### Hydrolase activity assays

Glucocerebrosidase and hexosaminidase activities were determined using the artificial substrates 4-methylumbelliferyl (4-MU)-β-D-glucoside or 4-MU-N-acetyl-β-D-glucosaminide as previously described (31). Briefly, twenty bodies without heads or 60 heads of 5-7-day-old flies were homogenized in 300 ul of KP buffer (50 mM potassium phosphate buffer, 0.25% Triton X-100, pH 6.5) and centrifuged at 4,000 g for 5 minutes. Supernatants were transferred to a fresh tube and assayed for the hydrolase activity in 0.1M sodium acetate buffer (pH 4.5). Lysates were incubated with Conduritol B epoxide (50 μM, 45 minutes, Toronto Research Chemicals, Inc) to subtract for non-lysosomal glucocerebrosidase mediated activity. Hydrolase reactions were stopped (1M Glycine-NaOH buffer, pH 12.5) and fluorescence quantified using a Spectra Max M2 fluorescent plate reader (excitation 365 nm, emission 445 nm, Molecular Devices). Protein levels were determined using the micro-BCA kit (Pierce, Rockford, IL). Each experiment was performed at least three times.

### Lifespan analysis

Longevity assays were conducted at 25°C. Groups of 10–20 age-matched flies were collected at 0–24 hours old and transferred to fresh standard food every 2–3 days. The number of dead flies was recorded during each transfer. Transfers were continued until all flies died. Kaplan-Meier lifespan curves were generated using Stata (StataCorp, College Station, TX), and analyzed by Cox proportional hazard models for statistically significant differences in survival between tested genotypes.

### Bang sensitivity assay

Vials containing 5 flies each were vortexed at maximum speed for 10 seconds, then observed until flies recovered. The time for full recovery with purposeful movements was recorded. Assays were videotaped for scoring. Recovery longer than 3 minutes was recorded as 3 minutes.

### Heat stress assay

Vials containing 10 flies each were submerged in a 39°C water bath for 6 minutes, then removed to room temperature and the time for full recovery with purposeful movements was recorded. Assays were videotaped for scoring. Recovery longer than 6 minutes was recorded as 6 minutes.

### Climbing assay

Climbing behavior was assayed as previously described [50]. Briefly, 20–30 flies were placed into the first chamber of a countercurrent apparatus, tapped to the bottom, and given 30 seconds to climb a distance of 10 cm. Flies that climbed 10 cm in 30 seconds were shifted to another chamber, and the experiment was repeated. After five trials, the flies in each chamber were counted, and the climbing index was calculated as the weighted average of the number of flies in each chamber divided by five times the number of flies in the assay.

### Learning and memory assay

Virgin females aged 13 days were placed with virgin males aged 13 days for 24 hours for mating. These females were then used as “trainers” for the subsequent conditioning assay. Naïve (virgin) 14-day-old males were placed with a recently mated “trainer” female for 1 hour in a conditioning/mating chamber. These conditioned males were returned to individual vials for 1, 6, or 24 hours, and then placed in a mating chamber with a freeze-killed virgin female as a courtship target and videotaped. A blinded investigator reviewed videos, and latency time was recorded as the number of seconds until the male extended one wing.

### Western blot analysis

30 day old whole flies or fly heads (8 females and 8 males) were homogenized in Triton Lysis Buffer (50 mM Tris-HCl pH 7.4, 1% Triton X-100, 150 mM NaCl, 1 mM EDTA), then spun at 15,000 rpm for 20 min. The Triton–soluble supernatant was collected. An equal volume of 2X Laemmli buffer (4% SDS, 20% glycerol, 120 mM Tris-Cl pH 6.8, 0.02% bromphenol blue, 2% β-mercaptoethanol) was added to the Triton–soluble supernatant, and the Triton–insoluble pellet was resuspended in 2X Laemmli buffer. All samples were boiled for 10 minutes. After boiling, the Triton–insoluble protein extracts were spun at 15,000 rpm for 10 minutes and the Triton–insoluble supernatant was collected. Triton–soluble and insoluble extracts were electrophoresed in 4–12% gradient acrylamide gels (Invitrogen) and transferred onto PVDF membranes. Membranes were blocked in 5% milk in 0.1% Tween/PBS and 0.1% normal goat serum. Immunodetections were performed using the following antibodies: 1:500 mouse anti-Ubiquitin (P4D4, Santa Cruz), 1:25,000 mouse anti-Actin (MAB1501, Chemicon/Bioscience Research Reagents), 1:1,000 mouse anti-human GBA1 (gift of Pablo Sardi, Sanofi Genzyme), 1:1,000 mouse anti-α-synuclein (610787, BD Biosciences), 1:200 mouse anti-Ref(2)P (Ab178440, Abcam). For immunoblotting of α-synuclein, PVDF membranes were fixed in 0.4% paraformaldehyde in PBS for 30 min after transfer, and then blocked in 5% milk/0.1% Tween/PBS before incubating with the α-synuclein antibody. Secondary antibody anti-mouse HRP (BioRad) was used at 1:10,000. Signal was detected using Thermo Scientific electrochemoluminescence reagents. Densitometry measurements of the western blot images were performed using Fiji software [67]. Normalized western blot data were log-transformed to stabilize variance before means were compared using Student *t* test. Each experiment was performed at least three times.

### Immunohistochemistry

30-day-old adult brains or thoracic muscle were dissected in cold Dulbecco’s Modified Eagle Medium (Sigma), and fixed in 4% paraformaldehyde/PBS for 30 min. Samples were washed in 0.1% Triton X-100/PBS. Fixed brains were stained with mouse anti-Tyrosine Hydroxylase (TH) antiserum Ab152 (1:100, Millipore) or mouse anti-poly Ubiquitin FK2 (1:200, Enzo), then anti-mouse Alexa 488 (1:200). Fixed muscle was also stained for anti-phalloidin-Alexa 568 (1:200) and DAPI (1:1000). Fixed tissues were mounted using ProLong Gold anti-fade medium (Molecular Probes).

### Image analysis

DA neurons were analyzed *in situ* as previously described [24]. An Olympus Fluoview-1000 confocal microscope was used to acquire optical sections of fixed brains at 1-μm intervals with identical parameters. The number of TH-positive neurons within the PPL1 dopaminergic neuron cluster was counted by visual inspection of individual confocal Z-series images by an investigator blinded to genotype.

For quantification of ubiquitin aggregates within thoracic muscle, each Z-stack of thoracic muscle was acquired with identical parameters (34 x 0.3 μm thick slices) using an Olympus Fluoview-1000 confocal microscope and deconvolved using Huygens Professional 14.10.0-2p4 software (Scientific Volume Imaging, Netherlands), with a signal to noise ratio of 20, maximum iterations of 140. The number of ubiquitinated protein aggregates per stack was calculated using the Advanced Object Analyzer of the Huygens software, using a threshold of 2 times the standard deviation of the image. The seed threshold for objects was set at 10% and the garbage threshold for objects at 35 voxels or smaller. Objects at the margins were also discarded, and watershed segmentation was used to differentiate between objects in close proximity to each other. At least 9 Z-stacks were analyzed per condition.

### Cathepsin D assay

The bioactivity of Cathepsin D was measured in 10 ng of protein from homogenized whole flies by a fluorescence-based assay using MCA-GKPILFFRLK(DNP)-dR-NH2 as a synthetic substrate (CTSD Activity Fluorometric Assay Kit; BioVision, Mountain View, CA). Fluorescence was measured at an excitation/emission = 328/460 nm using a Synergy H1 Hybrid Reader (BioTek, Winooski, VT). Each experiment was performed at least three times.

## Supporting Information

S1 Fig *dGBA1b* is the predominant *GBA1* homolog in heads, and is nearly undetectable in *GBA1*^*ΔTT*^ mutant heads. (A) qPCR primers used for relative quantification of *dGBA1a* and *dGBA1b* expression are indicated in dashed arrows. (B) Relative quantification of *dGBA1a* and *dGBA1b* expression by qPCR in the indicated adult tissues of 5-7-day-old control and *GBA1*^*ΔTT/ΔTT*^ homozygote flies. Error bars represent standard deviation, **p<0.005 by Student *t* test in all results shown in this figure.
(TIF)

S2 Fig *GBA1*^*ΔTT*^ results in a fusion transcript that is nearly undetectable in *GBA1*^*ΔTT*^ mutant heads. (A) The indicated qPCR primers flanking the breakpoints of *GBA1*^*ΔTT*^ were used to detect whether a fusion transcript was present. (B) PCR amplification from cDNA of *GBA1*^*ΔTT*^ homozygotes resulting from primers 1 and 3 in lane 1, and primers 2 and 4 in lane 2, indicating that a fusion transcript is present due to the *GBA1*^*ΔTT*^ allele. If no fusion transcript was present, no PCR product would be expected as Primer 1 and 3 would flank 4,457 bps, and primers 2 and 4 would flank 4,453 bps. (C) Relative quantification of the fusion transcript in *GBA1*^*ΔTT*^ homozygotes by qPCR in the indicated tissues. Error bars represent standard deviation, **p<0.005 by Student *t* test.
(TIF)

S3 Fig *CG31413* is only expressed in males, and the P-element insertion in *CG31413* does not reduce lifespan or cause behavioral phenotypes similar to those of *GBA1*^*ΔTT*^ mutants. (A) *CG31413* encodes a 561-amino-acid protein. The P-element Mi{ET1}*CG31413*^MB02698^ insertion site is predicted to interrupt the single exon of *CG31413* at amino acid position 119. (B) Relative quantification of expression of *CG31413* in adult WT males versus females. Error bars represent standard deviation. (C) Relative quantification of CG31413 expression by qPCR of the indicated genotypes. Error bars represent standard deviation. (D) Kaplan-Meier survival curves of *GBA1*^*+/+*^, *GBA1*^*ΔTT/ΔTT*^, and flies heterozygous for the P-element Mi{ET1}*CG31413*^MB02698^ insertion *in trans* to the *GBA1*^*ΔTT*^ allele (Mi{ET1}*CG31413*^MB02698^/*GBA1*^*ΔTT*^). (E) Climbing index of *GBA1*^*+/+*^, *GBA1*^*ΔTT/ΔTT*^, and Mi{ET1}*CG31413*^MB02698^/*GBA1*^*ΔTT*^ at the indicated ages. (F) Recovery time from mechanical stress (bang sensitivity) at the indicated ages in *GBA1*^*+/+*^ and Mi{ET1}*CG31413*^MB02698^/*GBA1*^*ΔTT*^. Error bars represent s.e.m. unless otherwise indicated, ns indicates *p*>0.05, *p<0.05, **p<0.005 by Student *t* test in all results shown in this figure.
(TIF)

S4 Fig Expression of Human WT GBA1 and Human GBA1 p.N370S in *Drosophila*. Western blot analysis using antiserum to human GBA1 of protein fractions from whole *GBA1*^*+/+*^ and *GBA1*^*ΔTT/ΔTT*^ flies ubiquitously expressing human WT GBA1 (*Act-G4>UAS-hGBA1*^*WT*^*; GBA1*^*+/+*^ and *Act-G4>UAS-hGBA1*^*WT*^*;GBA1*^*ΔTT/ΔTT*^) and human GBA1 p.N370S (*Act-G4>UAS-hGBA1*^*N370S*^*;GBA1*^*+/+*^ and *Act-G4>UAS-hGBA1*^*N370S*^*;GBA1*^*ΔTT/ΔTT*^) transgenes. Anti-β-Actin (βAct) was used as a loading control.
(TIF)

S5 Fig Ubiquitously expressed α-synuclein p.A53T does not significantly enhance *GBA1*^*ΔTT*^ mutant phenotypes. (A) Western blot analysis of Ub of Triton-insoluble protein fractions from heads of 30-day-old controls ectopically expressing human α-synuclein^*A53T*^ using the *Actin-GAL4* driver (*Act-G4>UAS-syn*^*A53T*^*;GBA*^*+/+*^) and *GBA1*^*ΔTT*^ homozygotes ectopically expressing human α-synuclein^*A53T*^ using the *Actin-GAL4* driver (*Act-G4>UAS-syn*^*A53T*^*;GBA1*^*ΔTT/ΔTT*^), with βAct loading control. (B) Densitometric quantification of Ub signal in the Triton-insoluble fraction from 30-day-old heads of flies of indicated genotypes as described in A. Levels of Ub signal per genotype were normalized to respective βAct loading controls, and these ratios were in turn normalized to the insoluble Ub/βAct ratio of *GBA1*^*+/+*^. *N =* 4. (C) Kaplan-Meier survival curves of lifespans of indicated genotypes as described in A. (D) Climbing index of 5-day-old flies of indicated genotypes as described in A. Error bars represent s.e.m., *p<0.05, **p<0.005 by Student *t* test for all results presented in this figure.SI_Caption>
(TIF)

S1 Table Summary of Lifespans in Fig 2A. (TIF)

S2 Table Summary of Lifespans in Fig 6A. (TIF)

S3 Table Summary of Lifespans in S3D Fig. (TIF)

S4 Table Summary of Lifespans in S5D Fig. (TIF)
