# Supplementary figures and images for: Glucocerebrosidase Deficiency in Drosophila Results in α-Synuclein-Independent Protein Aggregation and Neurodegeneration
Source: PLoS Genet. 2016 Mar 28;12(3):e1005944. doi: 10.1371/journal.pgen.1005944 (PMC4809718; doi:10.1371/journal.pgen.1005944)

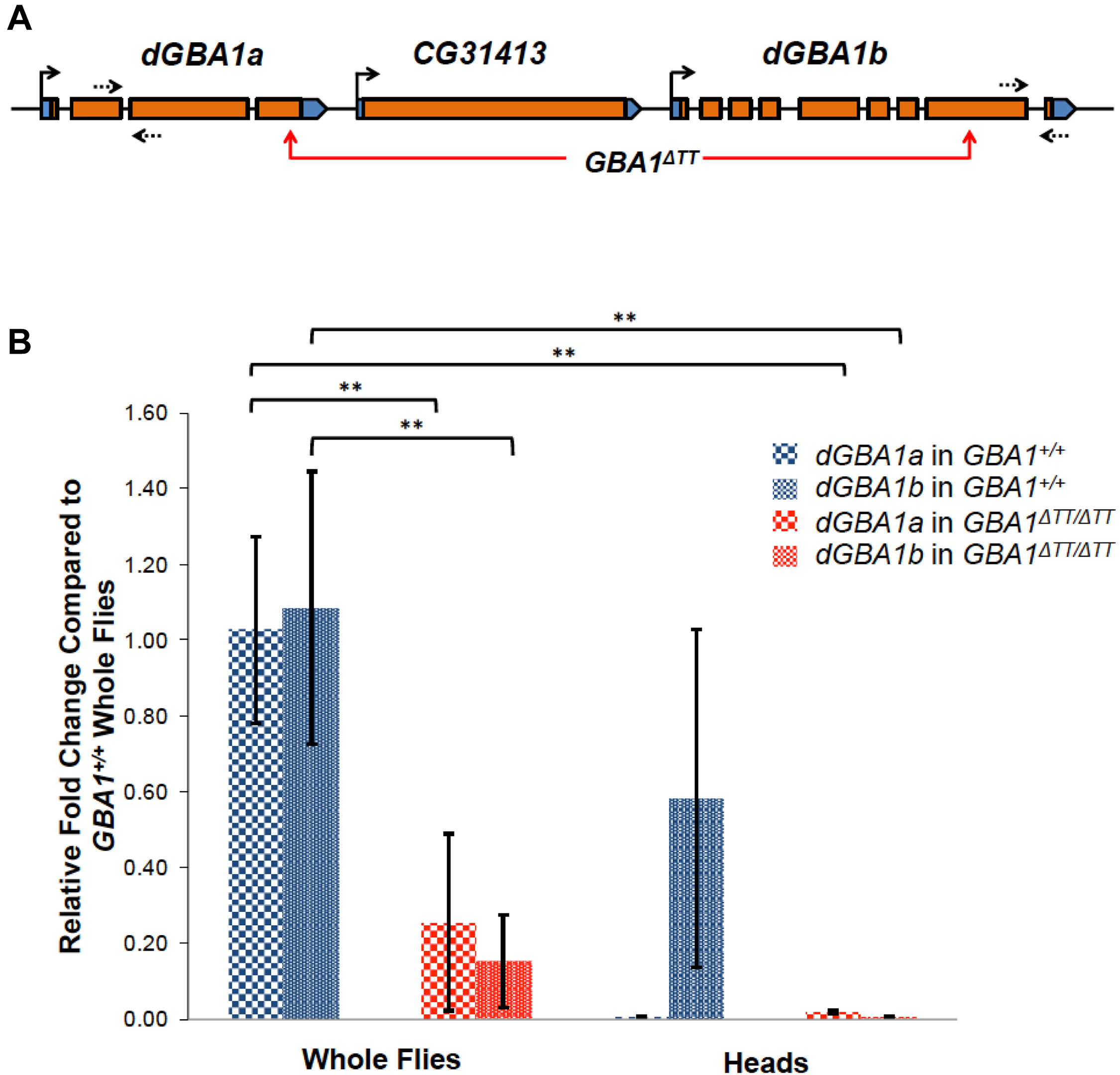

Supplement: S1 Fig — (A) qPCR primers used for relative quantification of dGBA1a and dGBA1b expression are indicated in dashed arrows. (B) Relative quantification of dGBA1a and dGBA1b expression by qPCR in the indicated adult tissues of 5-7-day-old control and GBA1ΔTT/ΔTT homozygote flies. Error bars represent standard deviation, **p<0.005 by Student t test in all results shown in this figure. (TIF) [file pgen.1005944.s001.tif]

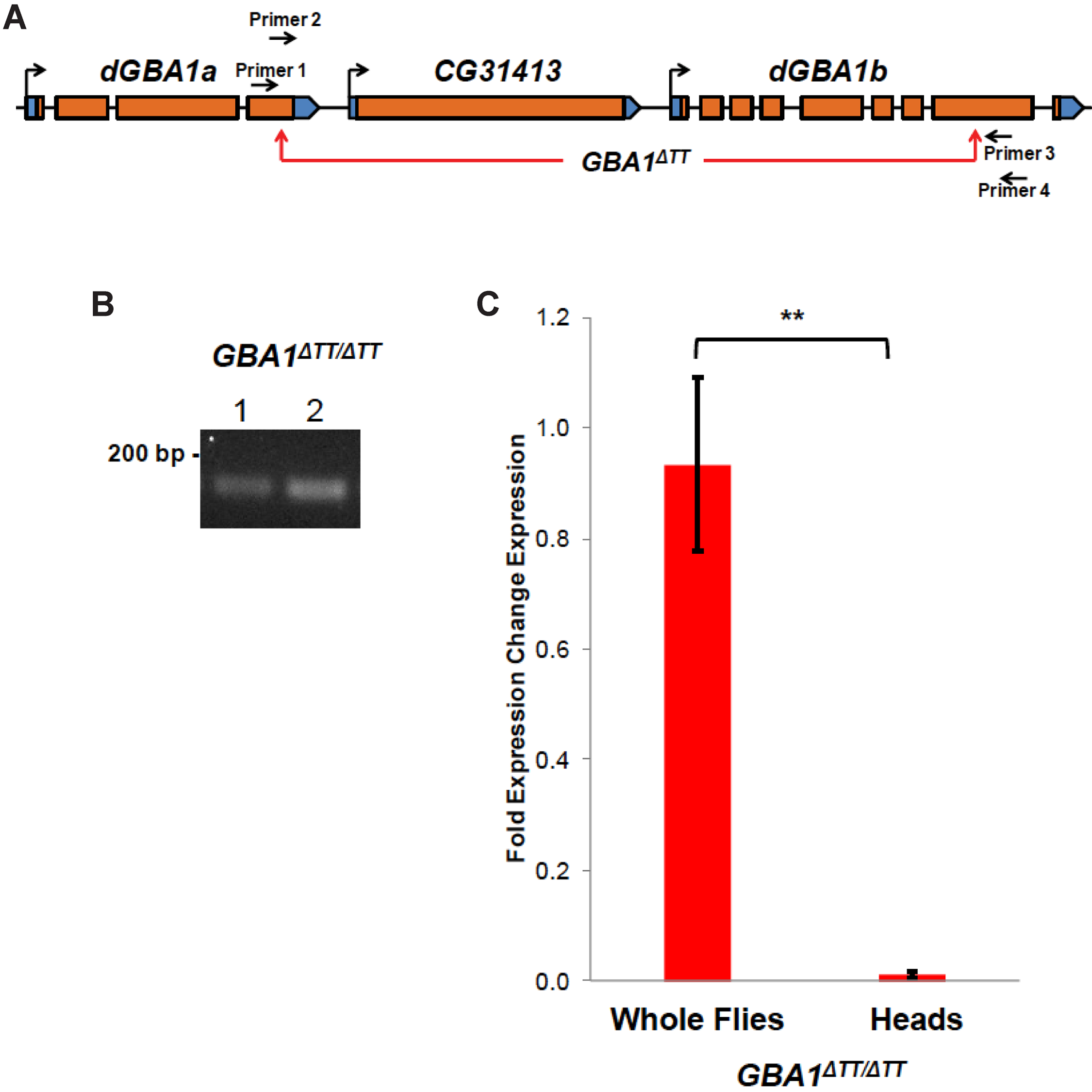

Supplement: S2 Fig — (A) The indicated qPCR primers flanking the breakpoints of GBA1ΔTT were used to detect whether a fusion transcript was present. (B) PCR amplification from cDNA of GBA1ΔTT homozygotes resulting from primers 1 and 3 in lane 1, and primers 2 and 4 in lane 2, indicating that a fusion transcript is present due to the GBA1ΔTT allele. If no fusion transcript was present, no PCR product would be expected as Primer 1 and 3 would flank 4,457 bps, and primers 2 and 4 would flank 4,453 bps. (C) Relative quantification of the fusion transcript in GBA1ΔTT homozygotes by qPCR in the indicated tissues. Error bars represent standard deviation, **p<0.005 by Student t test. (TIF) [file pgen.1005944.s002.tif]

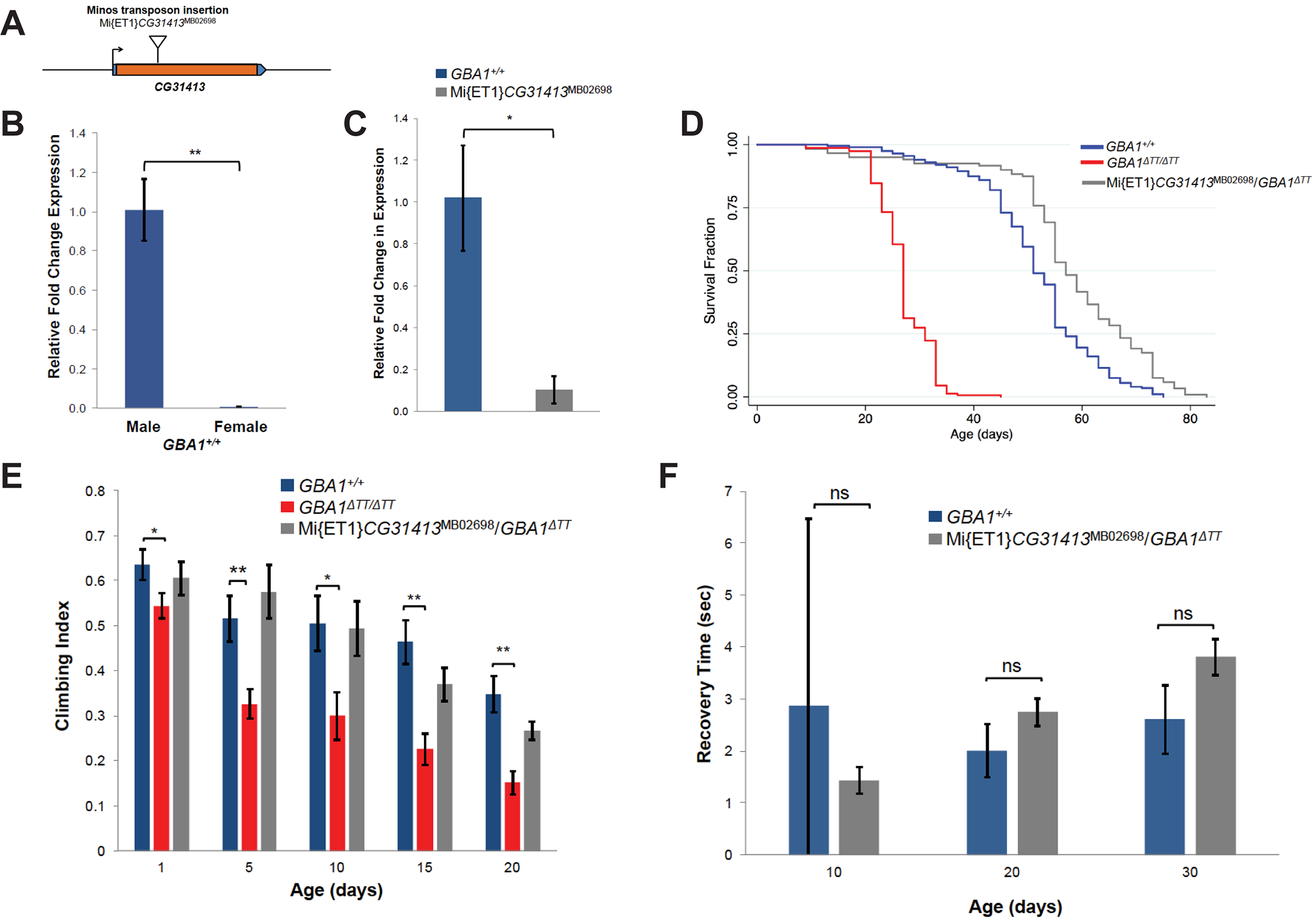

Supplement: S3 Fig — (A) CG31413 encodes a 561-amino-acid protein. The P-element Mi{ET1}CG31413MB02698 insertion site is predicted to interrupt the single exon of CG31413 at amino acid position 119. (B) Relative quantification of expression of CG31413 in adult WT males versus females. Error bars represent standard deviation. (C) Relative quantification of CG31413 expression by qPCR of the indicated genotypes. Error bars represent standard deviation. (D) Kaplan-Meier survival curves of GBA1+/+, GBA1ΔTT/ΔTT, and flies heterozygous for the P-element Mi{ET1}CG31413MB02698 insertion in trans to the GBA1ΔTT allele (Mi{ET1}CG31413MB02698/GBA1ΔTT). (E) Climbing index of GBA1+/+, GBA1ΔTT/ΔTT, and Mi{ET1}CG31413MB02698/GBA1ΔTT at the indicated ages. (F) Recovery time from mechanical stress (bang sensitivity) at the indicated ages in GBA1+/+ and Mi{ET1}CG31413MB02698/GBA1ΔTT. Error bars represent s.e.m. unless otherwise indicated, ns indicates p>0.05, *p<0.05, **p<0.005 by Student t test in all results shown in this figure. (TIF) [file pgen.1005944.s003.tif]

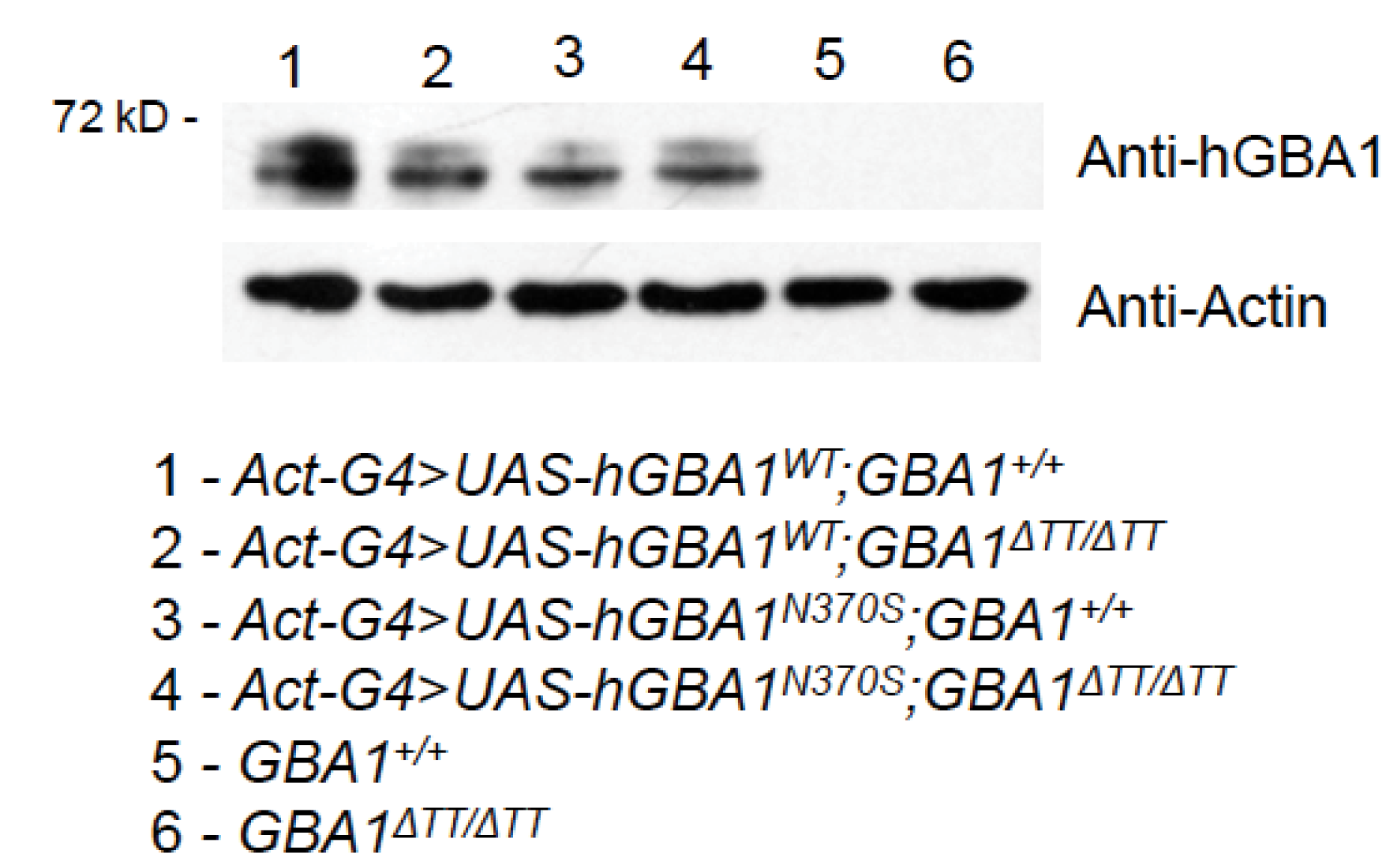

Supplement: S4 Fig — Western blot analysis using antiserum to human GBA1 of protein fractions from whole GBA1+/+ and GBA1ΔTT/ΔTT flies ubiquitously expressing human WT GBA1 (Act-G4>UAS-hGBA1WT; GBA1+/+ and Act-G4>UAS-hGBA1WT;GBA1ΔTT/ΔTT) and human GBA1 p.N370S (Act-G4>UAS-hGBA1N370S;GBA1+/+ and Act-G4>UAS-hGBA1N370S;GBA1ΔTT/ΔTT) transgenes. Anti-β-Actin (βAct) was used as a loading control. (TIF) [file pgen.1005944.s004.tif]

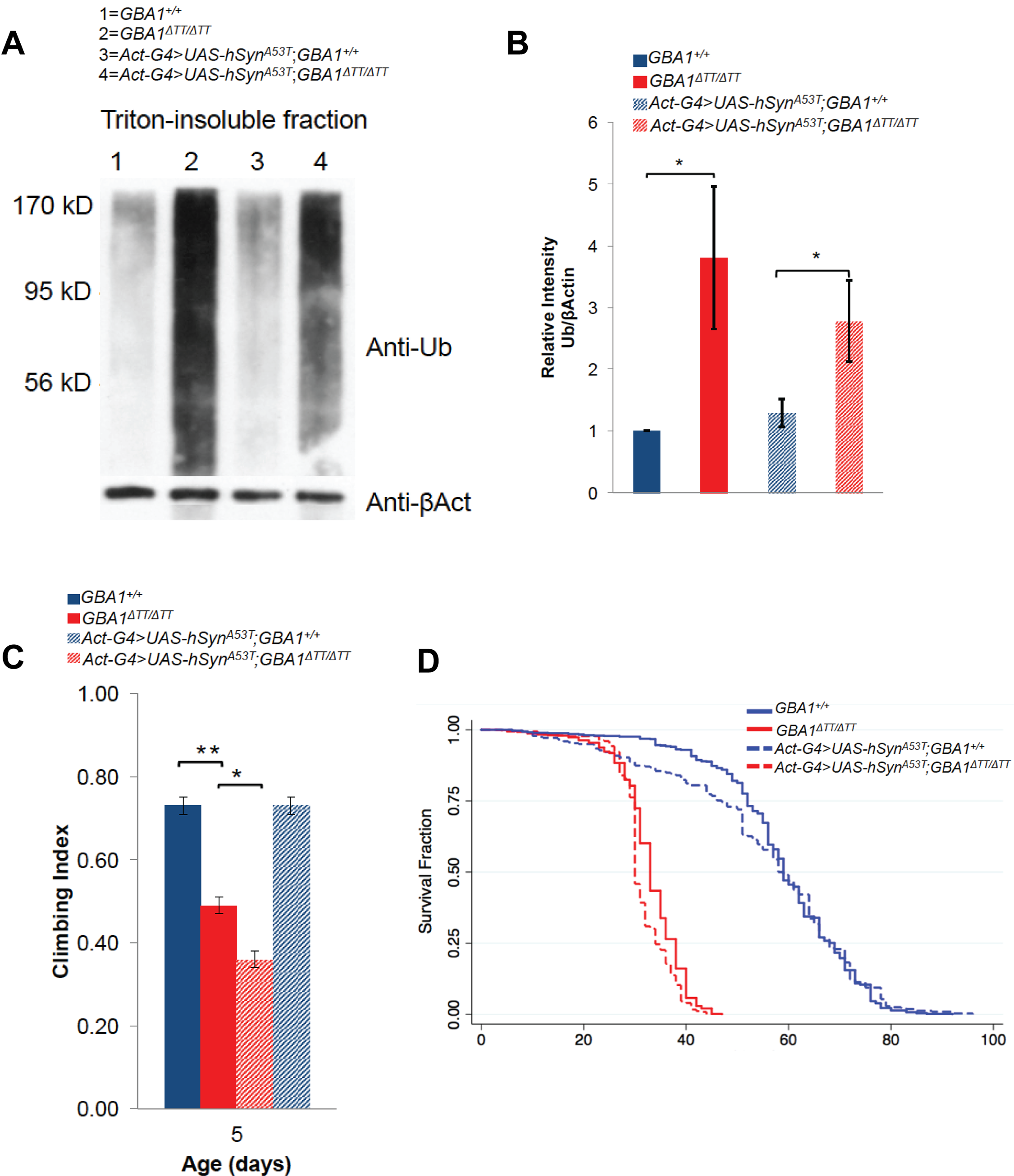

Supplement: S5 Fig — (A) Western blot analysis of Ub of Triton-insoluble protein fractions from heads of 30-day-old controls ectopically expressing human α-synucleinA53T using the Actin-GAL4 driver (Act-G4>UAS-synA53T;GBA+/+) and GBA1ΔTT homozygotes ectopically expressing human α-synucleinA53T using the Actin-GAL4 driver (Act-G4>UAS-synA53T;GBA1ΔTT/ΔTT), with βAct loading control. (B) Densitometric quantification of Ub signal in the Triton-insoluble fraction from 30-day-old heads of flies of indicated genotypes as described in A. Levels of Ub signal per genotype were normalized to respective βAct loading controls, and these ratios were in turn normalized to the insoluble Ub/βAct ratio of GBA1+/+. N = 4. (C) Kaplan-Meier survival curves of lifespans of indicated genotypes as described in A. (D) Climbing index of 5-day-old flies of indicated genotypes as described in A. Error bars represent s.e.m., *p<0.05, **p<0.005 by Student t test for all results presented in this figure.SI_Caption> (TIF) [file pgen.1005944.s005.tif]

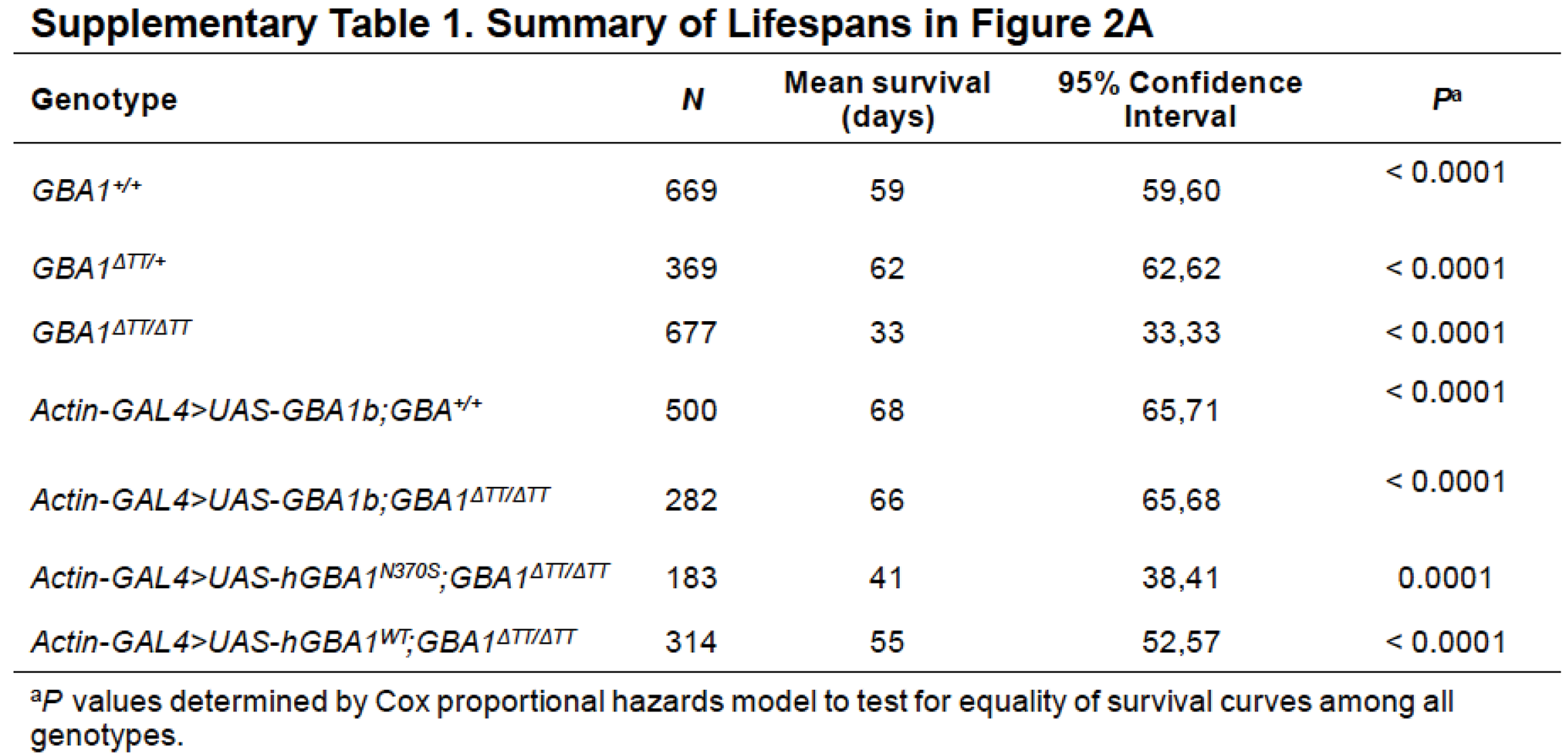

Supplement: S1 Table — (TIF) [file pgen.1005944.s006.tif]

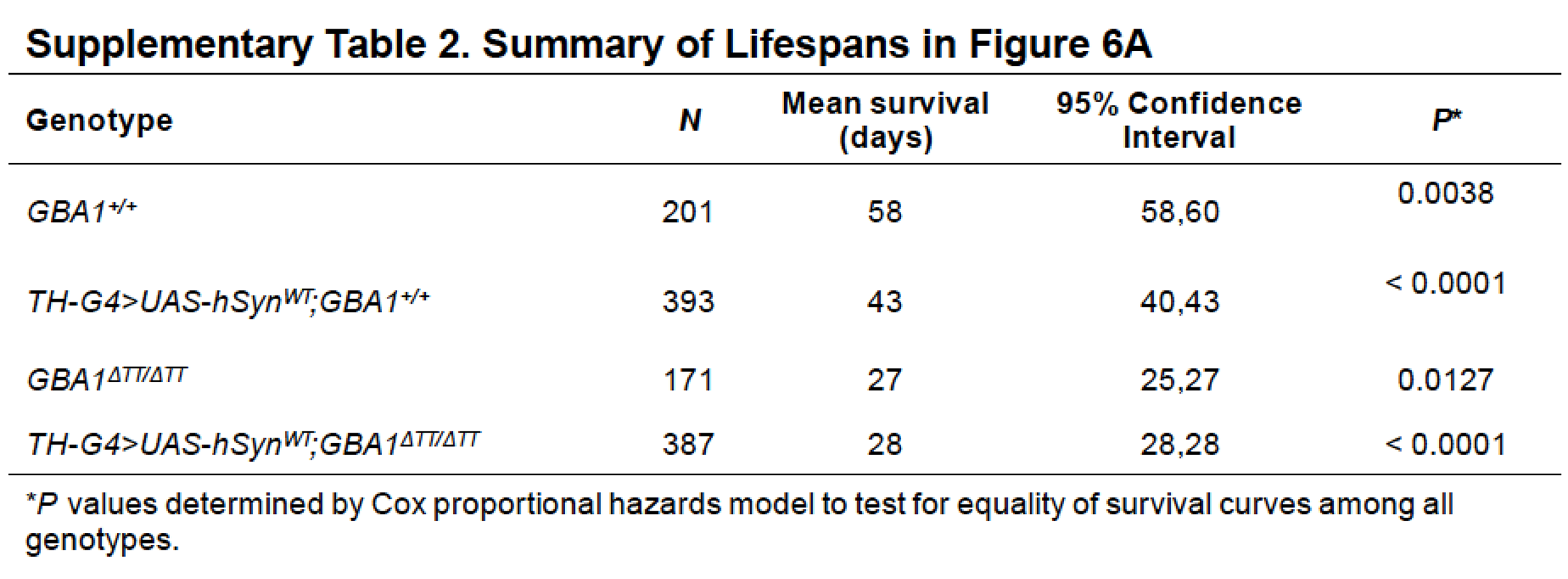

Supplement: S2 Table — (TIF) [file pgen.1005944.s007.tif]

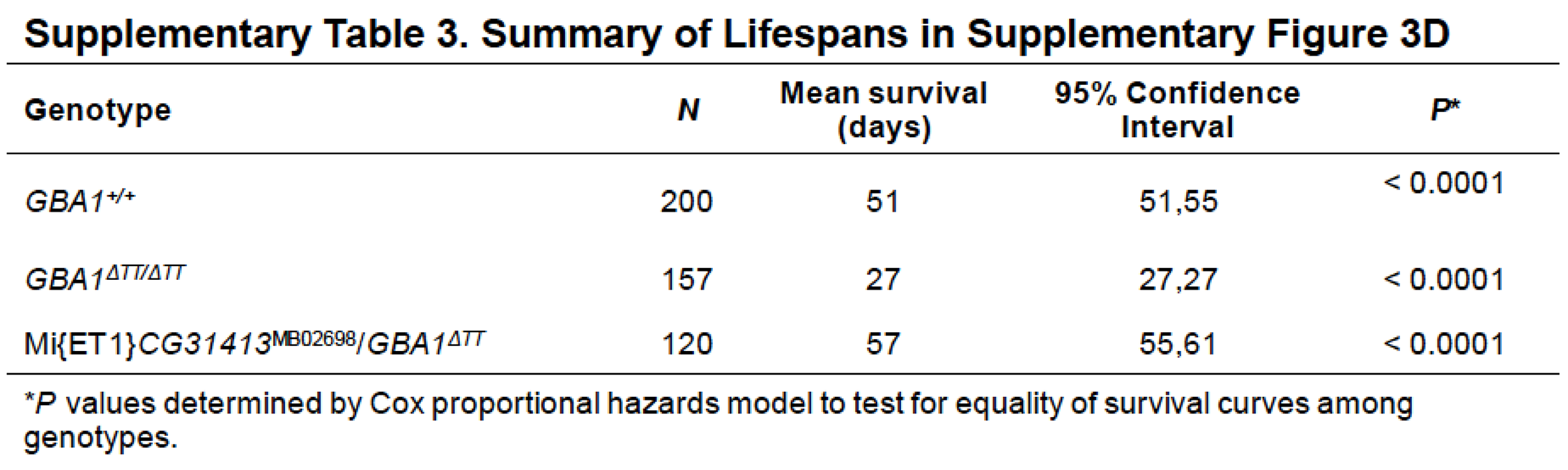

Supplement: S3 Table — (TIF) [file pgen.1005944.s008.tif]

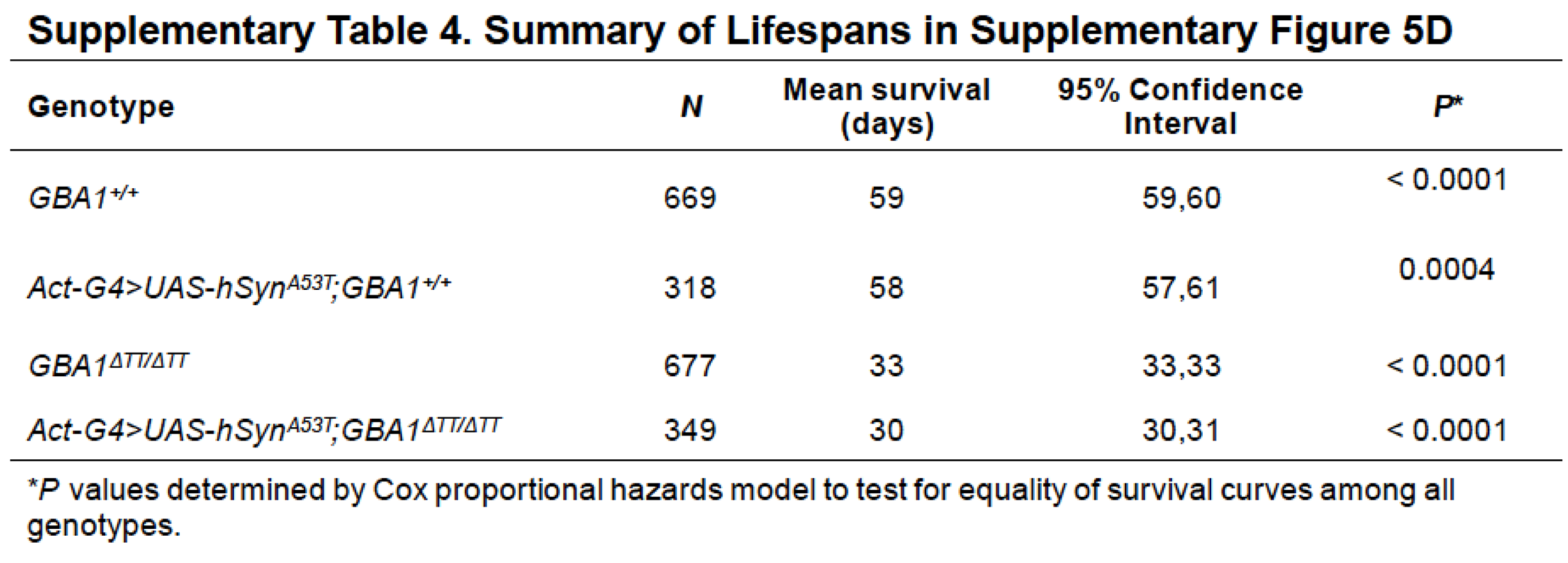

Supplement: S4 Table — (TIF) [file pgen.1005944.s009.tif]
